# Rhizobacterial syntrophy between a helper and a beneficiary promotes tomato plant health

**DOI:** 10.1093/ismejo/wrae120

**Published:** 2024-07-02

**Authors:** Sang-Moo Lee, Roniya Thapa Magar, Min Kyeong Jung, Hyun Gi Kong, Ju Yeon Song, Joo Hwan Kwon, Minseo Choi, Hyoung Ju Lee, Seung Yeup Lee, Raees Khan, Jihyun F Kim, Seon-Woo Lee

**Affiliations:** Institute of Agricultural Life Sciences, Dong-A University, Busan 49315, Republic of Korea; Department of Applied Bioscience, Dong-A University, Busan 49315, Republic of Korea; Department of Applied Bioscience, Dong-A University, Busan 49315, Republic of Korea; Department of Applied Bioscience, Dong-A University, Busan 49315, Republic of Korea; Department of Plant Medicine, Chungbuk National University, Cheongju 28644, Republic of Korea; Department of Systems Biology and Institute for Life Science and Biotechnology, Yonsei University, Seoul 03722, Republic of Korea; Department of Applied Bioscience, Dong-A University, Busan 49315, Republic of Korea; Department of Applied Bioscience, Dong-A University, Busan 49315, Republic of Korea; Department of Applied Bioscience, Dong-A University, Busan 49315, Republic of Korea; Department of Applied Bioscience, Dong-A University, Busan 49315, Republic of Korea; Department of Applied Bioscience, Dong-A University, Busan 49315, Republic of Korea; Department of Sciences, National University of Medical Sciences, Rawalpindi 46000, Pakistan; Department of Systems Biology and Institute for Life Science and Biotechnology, Yonsei University, Seoul 03722, Republic of Korea; Microbiome Initiative, Yonsei University, Seoul 03722, Republic of Korea; Institute of Agricultural Life Sciences, Dong-A University, Busan 49315, Republic of Korea; Department of Applied Bioscience, Dong-A University, Busan 49315, Republic of Korea

**Keywords:** bacterial wilt, Black Queen Hypothesis, helper-Beneficiary, microbial interactions, rhizosphere microbiota, succinate

## Abstract

Microbial interactions impact the functioning of microbial communities. However, microbial interactions within host-associated communities remain poorly understood. Here, we report that the beneficiary rhizobacterium *Niallia* sp. RD1 requires the helper *Pseudomonas putida* H3 for bacterial growth and beneficial interactions with the plant host. In the absence of the helper H3 strain, the *Niallia* sp. RD1 strain exhibited weak respiration and elongated cell morphology without forming bacterial colonies. A transposon mutant of H3 in a gene encoding succinate-semialdehyde dehydrogenase displayed much attenuated support of RD1 colony formation. Through the subsequent addition of succinate to the media, we found that succinate serves as a public good that supports RD1 growth. Comparative genome analysis highlighted that RD1 lacked the gene for sufficient succinate, suggesting its evolution as a beneficiary of succinate biosynthesis. The syntrophic interaction between RD1 and H3 efficiently protected tomato plants from bacterial wilt and promoted tomato growth. The addition of succinate to the medium restored complex II-dependent respiration in RD1 and facilitated the cultivation of various bacterial isolates from the rhizosphere. Taken together, we delineate energy auxotrophic beneficiaries ubiquitous in the microbial community, and these beneficiaries could benefit host plants with the aid of helpers in the rhizosphere.

## Introduction

Microbial interactions play an important role in maintaining the balance of complex communities by modulating microbial abundance and distribution. These interactions further influence the function of microbial communities and various ecosystems, which is of ecological importance [1, 2]. Negative interactions are predominant in various communities. For instance, various soil microbes compete for nutrients and antagonize each other through antibiosis [3]. However, there are cooperative interactions among microbial members through nutrient exchange, biofilm formation, and resistance to external attacks [4–7]. The Black Queen Hypothesis (BQH) proposes a unique cooperative interaction between helper-beneficiary in the microbial community [8]. Beneficiaries avoid the burden of certain functions by adapting to the environment through gene loss, resulting in subsequent genome reduction [8]. Thus, the beneficiary requires a certain function provided by the helper bacterium; i.e. they cannot multiply alone [8–12]. This process is important for long-lasting interactions, as beneficiaries develop a strong dependency on helpers [10]. Various cooperative interactions exist in nature, but validating these using isolated strains is challenging because of the predominance of competitive interactions and the unculturability of microbes [13, 14].

Syntrophic helper-beneficiary interactions have mainly focused on free-living microorganisms using culturable strains [11, 15–17]. One of the well-known examples is the reduction of oxidative stress in various marine heterotrophs through catalase, which facilitates the growth of *Prochlorococcus* [8, 9, 16]. A beneficiary that does not form bacterial colonies on standard media can be cultivated using siderophores produced from neighbor helpers [18]. Furthermore, the auxotrophy of nutrients, such as amino acids and vitamins, has been demonstrated to determine the syntrophic interactions between helper and beneficiary microbes [10, 16, 19]. Recently, computational prediction and genome-scale metabolic models have highlighted the importance of syntrophic interactions and auxotrophy in the host-associated microbiota [20–22]. Specific auxotrophy in plant-associated microbial communities has been predicted and identified, but the effects of the syntrophic helper-beneficiary interaction on host plants are not well understood [21, 23, 24].

Plants benefit from bacteria colonizing their rhizosphere for growth promotion [25, 26], stress tolerance [27], and defense against plant pathogens [28–31], and some rhizobacteria are biocontrol agents used for disease protection [28, 32]. Various bacterial traits are involved in the promotion of plant health; however, most of the beneficial rhizobacteria studied so far are culturable strains isolated using traditional culture techniques. During the last few decades, these strains have shown inconsistent activity under field conditions as bioprotectants and biostimulants for crop cultivation. To overcome this problem, the co-inoculation of culture collections that mimic natural microbiota, the so-called synthetic community (SynCom), has been considered [33, 34], but, owing to the unculturability of soil microbes [35–39], the absence of specific taxa still limits the general utility of SynCom as a model system for the functional analysis of microbiota. Thus, the use of vital nutrients or helper microbes that mimic the natural milieu could extend the cultivation of beneficiary microbes and reveal their roles in the microbiota.

Here, we aimed to explore unique syntrophic helper-beneficiary interactions in the plant rhizosphere. Specifically, we characterized succinate-mediated syntrophic interactions between uncultured beneficiary *Niallia* sp. RD1 and a helper *Pseudomonas putida* H3 isolated from the tomato rhizosphere. Syntrophic interactions between RD1 and H3 improved the health and growth of tomato plants. We also reported the evolution of unique beneficiaries in host-associated microbiota.

## Materials and methods

### Isolation and identification of helper and beneficiary bacteria from tomato rhizosphere

Tomato seeds (*Solanum lycopersicum* cv. Hawaii7996) were surface-sterilized by 70% ethanol for 1 min and 1% sodium hypochlorite solution for 15 min before finally being rinsed extensively in sterilized distilled water (SDW). The seeds were germinated in SDW for 7 days. The seedlings were planted in pots filled with upland soil from the Dong-A University Agricultural Experiment Station (N35.239°, E128.978°) mixed with nursery soil (Punong Co., South Korea; v/v 1:1). After 4 weeks, the soil tightly attached to roots was separated by vigorous shaking in a sterile diluent (4.25 g/L NaCl, 0.15 g/L KH_2_PO_4_, 0.3 g/L Na_2_HPO_4_, 0.1 g/L MgSO_4_, 0.05 g/L gelatin). The soil suspension was centrifuged at 5000 × *g* for 10 min to collect the rhizosphere soils. The serially diluted soil suspension was spread on R2A medium (MB cell, Seoul, South Korea) and cultured at 25°C and 30°C for several days.

A single colony of helper H3 was cultured by multiple streakings. To obtain a beneficiary RD1, RD1 cells in the vicinity of H3 were suspended in a diluent. The RD1 suspension was spread onto R2A medium, and H3 cells were then inoculated in the center of the medium, and the plates were incubated until RD1 colonies formed around the H3. To identify the isolated microorganisms, 16S rRNA gene sequencing was performed at Cosmo Genetech Co. (Seoul, South Korea) using the primer pairs 8F(5′-AGAGTTTGATCCTGGCTCAG-3′) and 1497R(5′-GGTTACCTTGTTACGACT-3′).

### Field emission scanning electron microscope (FE-SEM)

To investigate the morphological variations of RD1 cells depending on their proximity to H3, field-emission scanning electron microscopy (FE-SEM) was used. In order to prepare bacterial strains for FE-SEM analysis, a polycarbonate filter (25 mm diameter, 0.2 μm pore size, Whatman) was placed on R2A medium, in which 50 μl of RD1 suspension (OD_600_ = 0.5) was plated. The H3 were inoculated in the center of the plate using a sterile toothpick and cultured at 25°C for 5 days.

The filtrate with grown bacterial cells was allowed to incubate to fix in 1% osmium tetroxide (OsO_4_) in 0.1 M phosphate buffer for 1 h and gently washed three times with 0.1 M phosphate buffer for 10 min. The fixed bacterial cells on the filter were immersed by floating in a series of ethanol solutions (30%, 50%, 70%, 80%, and 90%) for 10 min, and finally 100% ethanol was used for 30 min followed by dehydration in a critical point dryer (E3000, SPI, Inc., West Chester, USA). The dehydrated samples were coated with platinum and observed by FE-SEM (JSM-6700F; JEOL, Tokyo, Japan) at 5 kV.

### Viability test of beneficiary bacteria

To investigate the viability of RD1, a BacLight RedoxSensor CTC Vitality Kit (Molecular Probes, OR, USA) was used. A suspension of RD1 (OD_600_ = 0.5) was inoculated onto R2A medium with or without H3 cells at 30°C for 2 days. Each of the differentiated microorganisms was recovered by suspending them in 100 μl of filtrated phosphate-buffered saline. The bacterial suspension was mixed with 100 μl of 50 mM CTC reagent and incubated in a dark condition for 30 min at 37°C. Further, to counterstain the living bacteria, 1 μl of 10 μM SYTO24 was added to the CTC-labeled mixture and incubated for 15 min at 37°C without exposure to light. Finally, the stained samples were observed under a confocal laser-scanning microscope (LSM510; Carl Zeiss, Germany). Red fluorescence was observed at absorption and emission wavelengths of 450 and 630 nm, respectively, whereas green fluorescence was observed at absorption and emission wavelengths of 490 and 515 nm, respectively.

### Effect of exogenous succinate and oxidative phosphorylation inhibitors on RD1 growth

To test the effect of succinate on RD1 growth, RD1 was cultivated in R2A medium containing 0.1%, 0.5%, 1.0%, and 2.0% sodium succinate dibasic hexahydrate (Sigma-Aldrich, MA, USA) adjusted pH 7.2 for 3 days at 30°C. To analyze the correlation between the concentration of succinate and RD1 growth, 10 μl of RD1 suspension (OD_600_ = 1.0) was dropped on R2A containing varying concentrations of succinate and incubated at 30°C. After 3 days, the RD1 colonies were suspended in 1 ml of SDW, and the OD_600_ value was measured using a spectrophotometer (Thermo Fisher Scientific, MA, USA).

To investigate whether succinate affects oxidative phosphorylation in RD1, RD1 was grown in R2A medium containing succinate and oxidative phosphorylation inhibitors, including the complex I inhibitor rotenone, the complex II inhibitor malonic acid, and the complex IV inhibitor sodium azide. Based on previous studies, 50 μg/ml rotenone, 1.0% malonic acid, and 10 mM sodium azide (all from Sigma-Aldrich, MA, USA) were added in R2A medium with 1.0% succinate [40–42].

### Whole genome sequencing and comparative genome analysis

The RD1 genome was sequenced using PacBio (RS II), and *de novo* assembly of the quality-filtered reads was performed utilizing the PacBio SMRT Analysis 2.3.0. at ChunLab, Inc. (Seoul, South Korea). The RD1 genome was compared to the reference genomes from the *Bacillus* and *Niallia* genera retrieved from the National Center for Biotechnology Information (NCBI) GenBank database (Supplementary Table S1). Prokka software was used to predict genes and annotate genomes [43]. Functional orthologs related to metabolic pathways were identified using annotations generated from eggNOG-mapper, which provides KEGG Orthology numbers applicable to KEGG Mapper [44, 45].

### *In planta* experiments for bacterial virulence and plant growth promotion assay

The tomato cultivar Zuiken (susceptible to bacterial wilt) was used for the bacterial virulence assays. Surface-sterilized seeds were germinated in SDW for 7 days. The tomato plants were germinated and grown in a plant growth room maintained at 28°C under a 14-h light/10-h dark cycle. The seedlings were planted in a pot containing 17 g of autoclaved nursery soil (121*°*C for 40 min, twice). After 3 weeks, the seedlings were treated with the RD1 and H3 suspensions or SDW (negative control). The detailed preparation method of bacterial suspension for *in planta* experiments is described in the supplementary methods. After 3 days, the plants were inoculated with SL341 and scored for disease symptoms for 14 days. Bacterial wilt disease severity was scored using the following formula: (number of wilted leaves/total number of leaves) × 100 (%).

Surface-sterilized seeds of the tomato cultivar Hawaii7996 were used to investigate tomato growth promotion by RD1 and H3. The surface-sterilized seeds were soaked in 5 ml of each bacterial suspension and SDW. After 7 days, seedlings were transferred to pots containing non-sterilized upland soil and treated with 5 ml of the respective inoculants. The plants were grown in the controlled plant growth room for 5 weeks. After that, the fresh and dry weights of the plants were measured. Each experiment included three biological replicates, each containing 10 plants.

### Estimation of RD1 and H3 population using quantitative PCR (qPCR)

After 0, 5, and 10 days post-inoculation (dpi) of RD1 and H3 suspension, rhizosphere soil was collected from Hawaii7996 and grown for 2 weeks at autoclaved nursery soil. The experiment was repeated four times, with two biological replicates per treatment, and the rhizosphere soil sample was combined from two technical replicates (*n* = 4). DNA was extracted from 200 mg of rhizosphere soil using the FastDNA SPIN for soil kit (MP Biomedicals, CA, USA) following the manufacturer’s instructions. Populations of RD1 and H3 were detected by qPCR using strain-specific primers targeting *rpoD* genes (Supplementary Table S2). qPCR was performed in a 10 μl reaction volume containing 10 ng soil DNA template, 5 μL of iQTM SYBR Green Supermix (Bio-Rad, CA, USA), 1 μl of each primer (10 pM), and HPLC-grade H_2_O (Fisher Scientific, PA, USA) using CFX Connect Optics Module Real-Time PCR System (Bio-Rad, CA, USA). The qPCR program were as follows: initial polymerase activation at 95°C for 3 min, followed by 30 cycles of denaturation at 95°C for 30 s, annealing at 60°C for 30 s, and extension at 72°C for 1 min. To generate a standard curve of the *rpoD* gene fragment, qPCR was performed using gDNA serially diluted from RD1 and H3.

### 16S rRNA gene amplicon sequence analysis using cultured bacterial cells

30 g of the tomato rhizosphere soil obtained from an upland field was suspended in 300 ml of 2.5 mM 2-(*n*-morpholino) ethanesulfonic acid (MES) monohydrate buffer at 30°C and 200 rpm for 1 h. Then, 100 μl of 1/100 diluted soil suspensions were inoculated on R2A medium with or without 1% succinate and incubated at 30°C. After 5 days, whole bacterial colonies on the selected plate were suspended in 1 ml SDW. The bacterial DNA was extracted from 500 μl of the cell suspension using CTAB method [46].

The V3-V4 region of 16S rRNA gene was amplified using dual indexed primers (341F,5′-**TCGTCGGCAGCGTCAGATGTGTATAAGAGACAG**CCTACGGGNGGCWGCAG-3′; 805R,5′-**GTCTCGTGGGCTCGGAGATGTGTATAAGAGACAG**GACTACHVGGGTATCTAATC-3′) with adapter overhang nucleotide sequences (bold text in the primers) [47, 48]. PCR was conducted using 2.5 μl of total DNA (5 ng/μl), 5 μl of each primer (1 μM), and 12.5 μl of the 2X KAPA HiFi Hotstart Ready Mix PCR Kit (Kapa Biosystems, MA, USA) following the manufacturer’s instructions. Amplified products were purified using AMPure XP beads (Beckman Coulter Genomics). Indexing PCR (2nd PCR) and DNA sequencing were conducted by NICEM (Seoul, South Korea) using an MiSeq platform (Illumina) based on the instructions provided, resulting in a set of paired end sequences of the 16S rRNA gene fragments. The 16S rRNA gene amplicons were analyzed using Quantitative Insights into Microbial Ecology 2 (QIIME2) version 2021.11 [49], following the available standard protocol, with slight modifications [50]. A detailed microbiome analysis pipeline with references is described in the supplementary methods.

### Statistical analysis

All the statistical details of the experiments can be found in the figure legends. Statistical analyses were performed using the R program and QIIME2.

## Results

### Identification of a beneficiary and a helper bacterium

**Figure 1:**
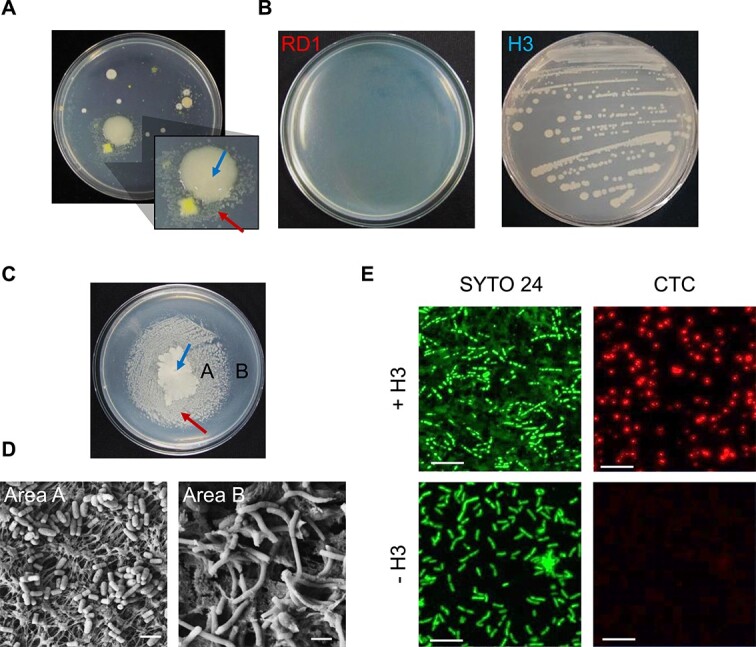
Syntrophic interaction between two bacterial strains isolated from tomato rhizosphere. (A) Satellite beneficiary bacteria (arrow below) RD1 having dense and small colonies were grown around a big colony of helper H3 bacteria (arrow above). (B) Growth of helper and beneficiary strains on R2A. Helper H3 strain formed a bacterial colony without RD1, but the beneficiary RD1 strain was not able to form a colony without H3. (C) Growth of RD1 (arrow below) was promoted by co-inoculation of H3 (arrow above). A, RD1 cells near to helper H3; B, RD1 cells far from H3. (D) Morphological observation of beneficiary RD1 that was used for FE-SEM. FE-SEM image of RD1 cells near helper H3 (Area A in Fig. 1C) or far from H3 (Area B in Fig. 1C). Scale bars of 1 μm are shown of each FE-SEM image. (E) Confocal laser scanning micrographs of bacterial cells after staining with CTC and SYTO 24. The red fluorescence from CTC staining (right panels) indicates CTC reduction, while the green fluorescence from SYTO 24 staining (left panels) indicates total bacterial cells. Scale bars, 10 μm. Area A: RD1 cells near to H3, Area B: RD1 cells far from H3.

We have previously isolated *Flavobacterium* sp. TRM1-10 from the rhizosphere soil of an upland field mesocosm, where it played a key role in conferring bacterial wilt resistance to tomatoes [30]. To expand the culture collection of tomato rhizosphere microbiota, we used the rhizosphere soil of tomato plants grown in upland soil. During the cultivation of bacterial isolates from the tomato rhizosphere soil suspension in R2A agar medium, a number of satellite bacterial colonies grew in the surroundings of large bacterial colonies (Fig. 1A). Satellite colonies were not cultured alone in the same medium (Fig. 1B); however, co-inoculation with the large central bacterial colony promoted the growth of satellite bacteria (Fig. 1C). The satellites and large bacterial colony were designated “RD1” and “H3,” respectively. The growth of RD1 was enhanced by its proximity to H3, but not on the I-plate, indicating that H3-derived diffusible substances might promote the growth of RD1 (Supplementary Fig. S1). RD1 and H3 were identified as *Niallia* sp. and *P. putida*, respectively, by comparing the 16S rRNA genes of RD1 and H3, and analyzing the whole genome of RD1. These results indicate that RD1 and H3 exhibit beneficiary-helper interactions.

### RD1 growth strongly depends on the presence of H3.

FE-SEM and bacterial viability assays were conducted to investigate the effect of H3 on the morphological plasticity and viability of RD1 (Fig. 1D and E). The colony of RD1 was observed within 2 cm of a drop of the H3 suspension (Fig. 1C). Within 2 cm of H3 (area A), RD1 cells appeared as short rods, whereas beyond 2 cm of H3 (area B), they exhibited a long rod or filament shape (Fig. 1C and D).

The viability of RD1 cells after co-inoculation with H3 was investigated using confocal laser scanning microscopy (Fig. 1E). The SYTO 24 stain, which fluoresces green and binds to nucleic acids in both live and dead cells, showed that the RD1 cells with H3 appeared rod-shaped, whereas those without H3 formed long-chain rod-shaped semi-filaments (Fig. 1C and E). Staining with 5-cyano-2,3-ditolyl tetrazolium chloride (CTC) indicated cell respiration and viability with red fluorescence. RD1 cells showed strong red fluorescence with H3, but weak fluorescence without H3 (Fig. 1C and E). These results indicate that the active growth and respiration of RD1 were determined by its proximity to H3.

### Growth of RD1 on standard media without helper

To investigate the growth-promoting factor for RD1, we tested 42 culture media containing different nutrients and substrates without helper cells (Supplementary Table S3). Initially, we inoculated a single RD1 strain on various standard media but observed no RD1 growth. The addition of diverse nutrients and supplements, including carbon sources (monosaccharides, disaccharides, propionate, and starch), nitrogen sources (tryptone and yeast extract), vitamin B (riboflavin, nicotinamide, and cyanocobalamin), ATP, catalase, antibiotics, heat-killed bacterial cells, detoxified medium, and the use of H3 on a cellulose membrane filter, failed to promote the growth of RD1 without helper cells.

### A transposon mutant of H3 alters syntrophic interaction

**Figure 2:**
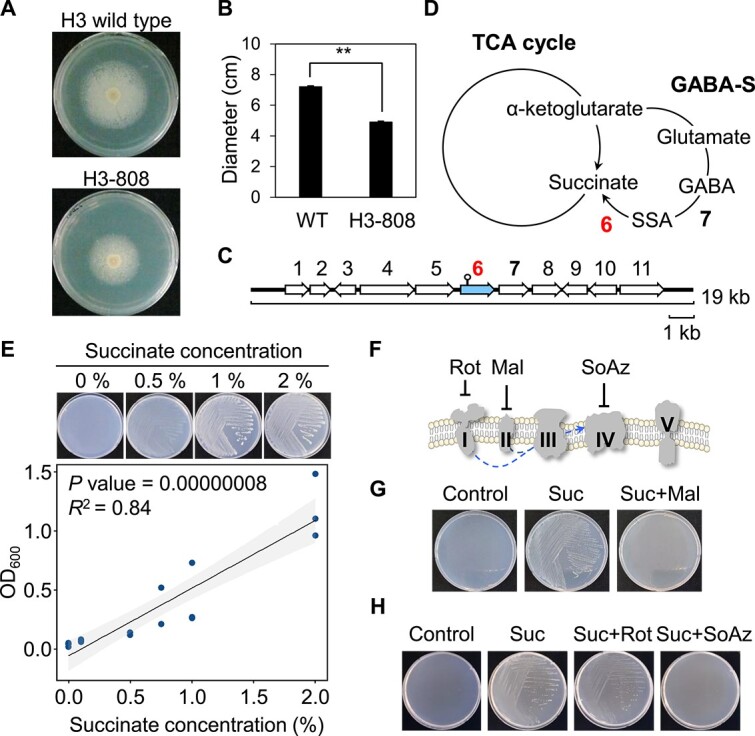
Transposon insertion in a gene encoding succinate semialdehyde dehydrogenase of H3 affects the syntrophic interaction between helper and beneficiary. (A, B) A mutant H3-808 strain exhibited the reduced syntrophic interaction with RD1 compared to H3 wild type. (C) Map of a 19 kb fragment of helper H3 strain carrying 11 ORFs encoding function of genes including Tn-inserted gene (gene 6) in H3-808. (D) A mutant H3-808 contained a Tn insertion in a gene encoding succinate semialdehyde dehydrogenase (*ssadh*) in GABA shunt pathway. 6, *ssadh* gene; 7, a gene encoding γ-aminobutyrate aminotransferase (*gabT*); GABA-S, GABA shunt pathway. (E) Exogenous succinate (>0.1%) promoted the colony formation of RD1 in R2A medium at 3 dpi (upper panels). The graph indicates the correlation between the concentration of succinate and the OD_600_ of RD1 colony (lower panel). (F) The inhibitors of electron transport chain systems. Rot, complex I inhibitor rotenone; Mal, complex II inhibitor malonate; SoAz, complex IV inhibitor sodium azide. (G) Malonate with the inhibition of RD1 colony formation in the presence of succinate. (H) Sodium azide inhibited the RD1 colony formation in the presence of succinate, but not rotenone. Control, R2A medium, negative control; Suc, R2A medium containing 1% succinate; Suc + Mal, R2A medium containing 1% succinate and 1% malonate; Suc + Rot, R2A medium containing 1% succinate and 50 μg/ml rotenone; Suc + SoAz, R2A medium containing 1% succinate and 10 mM sodium azide.

To identify helper-derived genes that promote RD1 growth, we screened more than 5000 transposon (Tn)-inserted H3 mutants. Among the H3 mutants, the Tn-mutant 808 (H3-808) showed a reduced diameter of the RD1 colony-forming area by 1.47-fold, compared with H3 wild-type (Fig. 2A and B). H3-808 contained a Tn insertion in the gene encoding NADP-dependent succinate-semialdehyde dehydrogenase (*ssadh*), which showed 100% similarity to the orthologous genes from *Pseudomonas* species (Fig. 2C and Supplementary Table S4). Ssadh facilitates succinate generation from diverse bacteria in the gamma-aminobutyric acid (GABA) shunt [51–55]. Mutation of the *ssadh* gene disrupts the oxidation of succinate semialdehyde to succinate in the GABA shunt and reduces succinate production in *Escherichia coli* and *Synechocystis* sp. [53, 56]. Based on the previous study, we hypothesized that succinate affects syntrophic interactions between RD1 and H3.

### Succinate-dependent growth and respiration of RD1

To validate the growth promotion of RD1 by exogenous succinate, RD1 was inoculated into R2A medium containing succinate without helper cells. Exogenous succinate significantly enhanced the growth of a single RD1 in R2A in a concentration-dependent manner (Fig. 2E). However, the other intermediates of the TCA cycle and GABA shunt, including citrate, α-ketoglutarate, malate, GABA, and L-glutamate, did not promote RD1 growth (Supplementary Fig. S2). Meanwhile, in minimal medium, succinate could only enhance RD1 growth with nitrogen sources, such as yeast extracts and protease peptone No. 3 (Supplementary Fig. S3). This indicated that succinate with a certain nitrogen source can support the growth of RD1 without helper.

Succinate is an electron donor to succinate dehydrogenase (complex II) in the electron transport chain [57]. To test whether exogenous succinate affects the oxidative phosphorylation of RD1, RD1 was inoculated onto R2A containing succinate and an inhibitor of respiration. The inhibitors included rotenone, malonate, and sodium azide, which inhibit NADH dehydrogenase (complex I), complex II, and cytochrome c oxidase (complex IV), respectively, in oxidative phosphorylation (Fig. 2F). Treatment with malonate and sodium azide, but not rotenone, completely inhibited the succinate-dependent growth of RD1 (Fig. 2G and H). These results indicate that exogenous succinate stimulates RD1 growth by activating complex II-dependent respiration.

### RD1 lacks genes involved in succinate production

Previously, plant-associated *Niallia* (previously *Bacillus* [58]) species have been readily cultured in standard medium [59, 60]. To compare RD1 with other plant-associated *Niallia* species, we sequenced the whole genome of RD1 and conducted a genome-wide comparative analysis of RD1 and other *Niallia* strains. The complete genome sequence of RD1 was deposited in GeneBank (Supplementary Table S1 and Supplementary Fig. S4). For genome comparison, we selected *Bacillus subtilis* strain 168 (Bs168) and four different plant-associated *Niallia* reference strains, including *N. circulans* GN03, PK3-109, PK3-15, and PK3-138 [59, 60] (Supplementary Table S1). The total genome length and the mean average GC content of RD1 are 5 394 080 bp and 35.65%, respectively, similar to other *Niallia* genomes (average length 5.09 Mbp, mean GC content 35.5%) (Supplementary Table S1).

**Figure 3:**
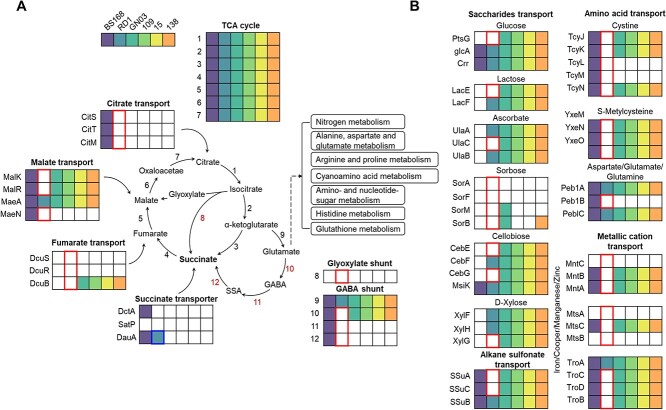
Thick-marked boxes indicate genes that are absent in the RD1 genome compared to other *Niallia* strains, except for the gene encoding the succinate transporter DauA. This gene is specifically present in the RD1 genome. (A) Loss of gene involved in tricarboxylate transporters, glyoxylate shunt, GABA shunt pathway and (B) ABC transporters for nutrient import in RD1. 1–7: TCA cycle related genes, 8: isocitrate lyase, 9: glutamate synthase, 10: glutamate decarboxylase (gad), 11: GABA transaminase (GabT), 12: succinate semialdehyde dehydrogenase (Ssadh), BS168, *B. subtilis* strain 168; RD1, *Niallia* sp. RD1; GN03, *Niallia circulans* GN03; 109, *N. circulans* strain PK3_109; 15, *N. circulans* strain PK3_15; 138, *N. circulans* strain PK3_138.

Succinate can be produced in bacteria via the GABA shunt, TCA cycle, and glyoxylate shunt [57]. In the GABA shunt, RD1 lacked the genes encoding glutamate decarboxylase (*gad*), GABA transaminase (*gabT*), and *ssadh* (Fig. 3A). Meanwhile, four *Niallia* strains showed a loss of *gabT* and *ssadh*, but not *gad*. Unexpectedly, there were no genetic differences between RD1 and the other strains in the TCA cycle and glyoxylate shunt; however, RD1 lacked genes encoding importers of the TCA cycle substrates, such as citrate (*citS*, *citT*, and *citM*), fumarate (*dcuS*, *dcuR*, and *dcuB*), and malate (*malK*, *malR*, and *maeN*), affecting the TCA cycle and succinate production. RD1 uniquely possesses the *dauA* gene, which encodes a succinate transporter, compared with other *Niallia* species. Furthermore, RD1 showed a loss of ABC transporters for saccharides, amino acids, sulfonates, and metallic cations (Fig. 3B). RD1 also lacked the antibiotic efflux pump for cationic antimicrobial peptides, bacitracin, lantibiotics, vancomycin, and irinotecan (Supplementary Fig. S5). Taken together, these data suggest that loss of function might cause succinate auxotrophy, and accordingly, respiratory deficiency, in RD1.

### Combination of RD1 and H3 affects plant health in tomato plant

**Figure 4:**
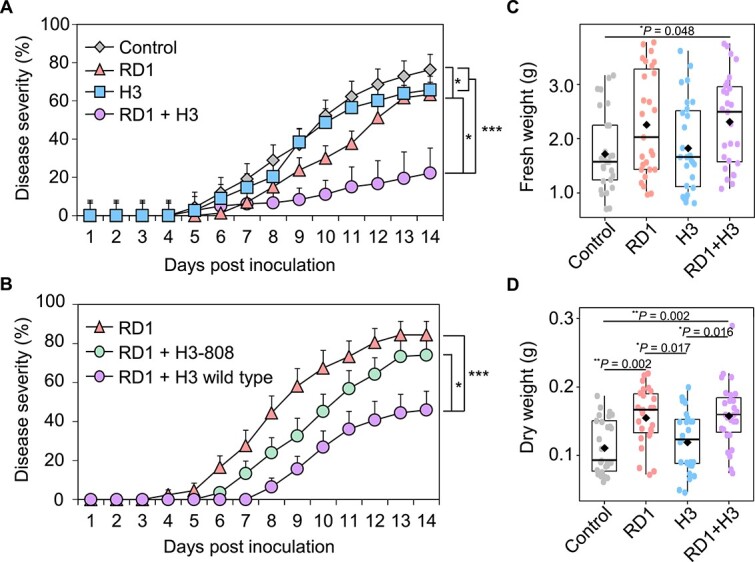
Co-inoculation of RD1 and H3 promotes tomato health. (A) Disease severity of bacterial wilt disease was measured for 14 dpi of *Ralstonia pseudosolanacearum* SL341 in Zuiken tomato treated with RD1, H3, and mixture. Zuiken tomato plants were treated with RD1, H3, and mixture of H3 and RD1 3 days before *R. pseudosolanacearum* SL341 inoculation. (B) Combination of H3-808 mutant and RD1 showed the reduction of disease control activity compared to mixture of H3 wild type and RD1. Values are the average of three replicates (each replication with 10 plants, *n* = 30 for each treatment) and vertical bars represent standard error. Significant difference was noticed by repeated measures ANOVA (^*^P < .05, ^*^^*^P < .01, ^*^^*^^*^P < .001). (C) Fresh weight and (D) dry weight of tomato plant were examined at 5 weeks after transplantation. Values are the average of three replicates (each replication with 9 plants, *n* = 27 for each treatment) and vertical bars represent standard error. Significant difference was noticed by Kruskal–Wallis test with Dunn’s *post hoc* test (^*^P < .05, ^*^^*^P < .01, ^*^^*^^*^P < .001). Diamonds and bolded lines of the boxplot are the average and median of indicated values, respectively. Control, S; RD1, the single inoculation of RD1; H3, the single inoculation of H3; RD1+H3; the co-inoculation of RD1 and H3. RD1+H3-808, the mixture of RD1 and H3-808 mutant; RD1+H3 wild type, the mixture of RD1 and H3 wild type.

As RD1 and H3 were isolated from the rhizosphere soil, we hypothesized that syntrophic interactions between rhizobacteria would benefit host plants. To test this hypothesis, we investigated the protective effect of RD1 and H3 against bacterial wilt caused by *Ralstonia pseudosolanacearum* SL341 and the tomato growth promotion in the presence of RD1 and H3 (Fig. 4). In plants treated with SDW (control), wilt symptoms began to appear at 5 dpi, and the disease severity of bacterial wilt was 76% at 14 dpi (Fig. 4A). The mixture of RD1 and H3 reduced disease severity by 3.45-, 3.00-, and 2.86-fold at 14 dpi compared to the control, RD1 alone, and H3 alone, respectively (Fig. 4A). The population of SL341 in tomato stems was reduced by the mixture of RD1 and H3 compared with the other treatments, whereas the mixture did not inhibit the growth of SL341 (Supplementary Figs S6 and S7). Furthermore, co-inoculation of RD1 and H3-808 mutants did not show biocontrol activity against SL341 compared to the combination of RD1 and H3 wild-type (Fig. 4B).

The combination of RD1 and H3 promoted tomato plant growth. Compared with the control, the mixed treatment of RD1 and H3 increased the fresh and dry weights of tomatoes by 1.34- and 1.41-fold at 5 weeks post-transplantation, respectively (Fig. 4C and D). Meanwhile, a single treatment with RD1 also enhanced the dry weight of tomatoes by 1.39-fold (Fig. 4D). Furthermore, at the early developmental stage, the combination of RD1 and H3 and a single treatment with H3 promoted shoot growth in tomato seedlings (Supplementary Fig. S8). Taken together, these data indicated that the combination of RD1 and H3 enhanced the growth and health of tomato plants.

### Syntrophy enhances the colonization of RD1 and H3 *in planta*

**Figure 5:**
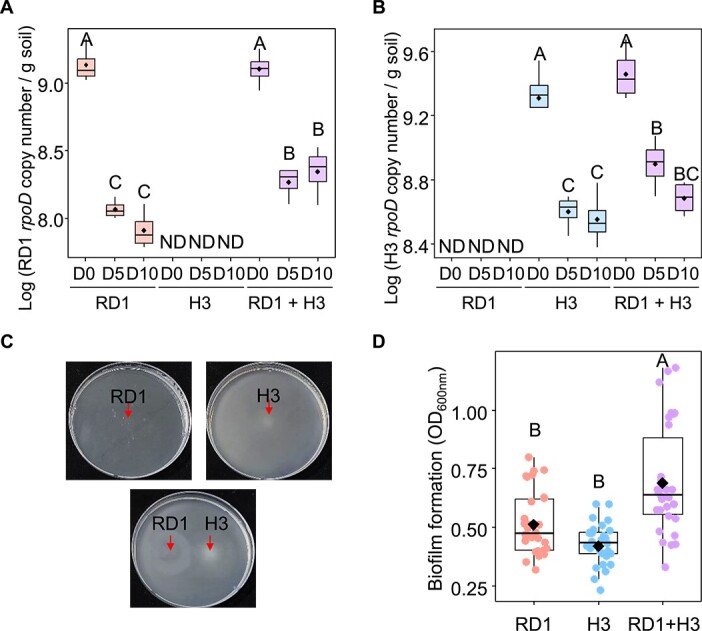
Syntrophic interaction enhances the colonization of RD1 and H3 *in planta*. (A, B) Colonization of RD1 and H3 in tomato rhizosphere soil. The bacterial population of (A) RD1 and (B) H3 in tomato rhizosphere soil were estimated with the RD1 or H3-specific primers targeting the *rpoD* genes using qPCR. Different letters on the box plot represent significant difference among means of the rhizosphere soil (*n* = 4) of each treatment by Tukey’s multiple range test. D0, 5, and 10 are 0, 5, and 10 dpi. (C) Swimming motility of RD1 and H3. The RD1 did not showed the swimming motility without H3, but H3 showed high swimming motility regardless of RD1 (upper panels). Swimming motility of RD1 was promoted by the co-inoculation of H3 (lower panel). The arrow indicates inoculation site of each bacterial cell suspension. (D) Biofilms were quantified as absorbance at 600 nm (OD_600_) following crystal-violet staining method using 96-well PVC microplate. The experiment were conducted in triplicate with 10 wells per treatment (*n* = 30 wells). Different letters on the box plot represent significant difference among means of each treatment by Tukey’s multiple range test. Diamonds and bolded lines of the boxplot are the average and median of indicated values, respectively. RD1, the single inoculation of RD1; H3, the single inoculation of H3; RD1+H3; the co-inoculation of RD1 and H3.

To validate the syntrophic interaction of RD1 and H3 *in planta*, the populations of RD1 and H3 in tomato rhizosphere soil were investigated using quantitative real-time PCR (qPCR) with strain-specific primers targeting the *rpoD* gene encoding the RNA polymerase sigma factor (Supplementary Fig. S9). The RD1 population remained over 10^8^ copies/g of rhizosphere soil in the presence of H3 at 5 and 10 dpi, whereas that of RD1 without H3 gradually declined to below 10^8^ copies/g of soil (Fig. 5A). Furthermore, the H3 population was decreased rapidly over time in the absence of RD1 (Fig. 5B). These results indicate that RD1 and H3 exhibit syntrophic interactions that maintain their populations *in planta*.

Cell motility and biofilm production are important traits for root colonization by rhizobacteria [61–63]. The syntrophic interaction between RD1 and H3 improved swimming motility and biofilm formation (Fig. 5C and D). The swimming motility of RD1 cells was particularly enhanced by co-inoculation with H3, but not without helper cells (Fig. 5C). Meanwhile, H3 exhibited high swimming motility independent of RD1 (Fig. 5C). In contrast, biofilm formation in the mixture of RD1 and H3 increased 1.35- and 1.63-fold, compared to individual RD1 or H3, respectively (Fig. 5D). Similarly, exogenous succinate promoted swimming motility and biofilm formation of RD1 *in vitro* (Supplementary Fig. S10). This suggests that the syntrophic interaction might enhance the colonization of RD1 and H3 via the activation of cell motility and biofilm formation *in planta*.

### Wide distribution of succinate auxotrophy in the rhizosphere

**Figure 6:**
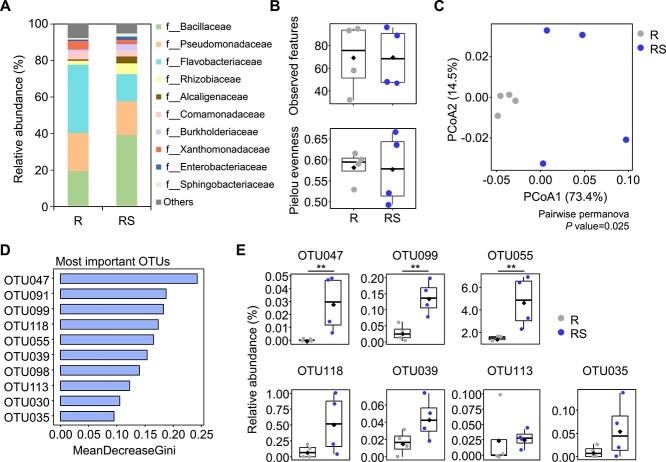
Exogenous succinate enhances the viability of diverse bacteria in soil microbiota. (A) Relative abundance of soil-derived bacterial colonies grown in R2A or R2A containing 1% succinate. (B) Alpha diversity of soil-derived bacteria l colonies grown in R2A or R2A containing 1% succinate. Diamond and the bolded lines in the boxplot are the average and median of alpha-diversity indices, respectively. (C) Two-dimensional principal coordinate analysis (PCoA) ordination based on the weighted UniFrac distance metric. (D) The top 10 differential OTUs between treatments selected by the random forest analysis. (E) The relative abundance of seven signature OTUs enriched by succinate treatment. The mean values ± SEs are given (*n* = 4). Significant difference was noticed by Student’s *t*-test (^*^P < .05, ^*^^*^P < .01, ^*^^*^^*^P < .001). Diamond and the bolded lines in the boxplot are the average and median of relative abundance of OTUs, respectively. R, the soil bacterial colonies grown at R2A agar medium; RS, the soil bacterial colonies grown at R2A agar medium with 1% succinate.

We hypothesized that succinate auxotrophy is ubiquitous under natural soil conditions. Therefore, we analyzed the microbiome of viable bacterial colonies grown in succinate-containing R2A medium using 16S rRNA gene amplicon sequencing to assess the influence of succinate on the rhizosphere microbiota of tomato plants grown under outfield conditions (Fig. 6). Exogenous succinate did not significantly increase the number of bacterial colonies (Supplementary Fig. S11). A total of 722 553 sequences were obtained from all samples, and which were clustered into 139 OTUs with 97% similarity. The rarefaction curves for the samples indicated near saturation of the number of observed OTUs (Supplementary Fig. S12). A comparison of the relative abundance of OTUs showed that exogenous succinate enriched OTUs in the following families compared with the control: *Bacillaceae*, *Rhizobiaceae*, *Alcaligenaceae*, *Burkholderiaceae*, *Enterobacteriaceae*, and *Sphingobacteriaceae* (Fig. 6A). Succinate treatment did not affect the richness and evenness indices of bacterial colonies (Fig. 6B), but principal coordinate analysis, based on the weighted UniFrac dissimilarity, showed distinct clusters between succinate-treated and control samples (Fig. 6C). The PCoA1 and PCoA2 axes accounted for 73.4% and 14.5% of the variance, respectively.

To identify the bacterial OTUs influenced by succinate, we obtained the top 10 differential OTUs using random forest analysis (Fig. 6D and E and Supplementary Table S5). The 10 OTUs included members of *Bacillaceae* (OTU047, 118, and 030), *Rhizobiaceae* (OTU055 and 035), *Microbacteriaceae* (OTU099), *Streptomycetaceae* (OTU039), *Sphingobacteriaceae* (OTU113), *Oxalobacteraceae* (OTU091), and *Brevibacillaceae* (OTU098) (Supplementary Table S5). Seven OTUs (OTU047, 099, 055, 118, 039, 113, and 035) were enriched by succinate, with six showing at least a 2-fold increase except for OTU113 (Fig. 6E). In addition, the abundances of six OTUs (OTU047, 099, 118, 039, 113, and 035) were lower than 0.1% without succinate (Fig. 6E). However, OTU091, 098, and 030, which decreased by succinate, had abundances above 0.1% without succinate (Supplementary Fig. S13). These results suggest that exogenous succinate stimulates the viability of rare taxa and that succinate auxotrophy may be ubiquitous in the natural rhizosphere microbiota.

## Discussion

Microbial cooperation plays an important role in microbiota function. BQH, a unique microbial cooperation, has been proposed for the life style of free-living microbes [8, 9, 15]; however, recent studies have predicted helper-beneficiary interactions in host plant-associated microbiota [21–24]. Here, we characterized the syntrophic interaction between the helper *P. putida* H3 and the beneficiary *Niallia* sp. RD1, isolated from the tomato rhizosphere [30]. Bacterial features of the RD1 in the aid of H3 fit to the concept of BQH. We found that syntrophic interactions benefit host plants by promoting plant growth and health. The beneficiary RD1 evolved into a succinate auxotroph by discarding succinate biosynthesis-related genes from its genome.

Syntrophic interactions in the BQH rely on helper-derived nutritionally or energetically valuable public goods shared by both helper and beneficiary [8, 9, 15, 24]. RD1 cells without H3 displayed a specific dormant state with weak respiration and cell elongation (Fig. 1D and E). This dormancy of RD1 was recovered by H3-derived succinate, demonstrating a syntrophic interaction between the helper and succinate-auxotrophic beneficiary (Fig. 2 and Supplementary Fig. S2). RD1 showed a preference for complex II-dependent respiration using succinate as an electron donor, despite its lower ATP production efficiency [57] (Fig. 2). This preference may explain the avoidance of cell damage because complex I-based respiration generates harmful reactive oxygen species that complex II does not leak [64]. The lack of efflux pumps for toxic compounds might render RD1 more vulnerable to stress (Supplementary Fig. S5). Our results provide a new perspective on energy auxotrophy in soil microbiota, distinct from conventional auxotrophy for nutrient resources or detoxifying compounds [8–10, 15, 16, 18, 19]. However, energy auxotrophy alone may be insufficient to explain the dynamic interactions among microorganisms in nature [19]. Indeed, RD1 exhibited co-auxotrophy to succinate and nitrogen resources (Supplementary Fig. S3), indicating the complex multiple metabolic dependencies of syntrophic interactions in the natural microbiota [15, 19, 65].

Genome comparisons show that gene loss is specific to succinate metabolism in RD1. RD1 showed loss of function in succinate biosynthesis and ABC transporters for diverse nutrients (Fig. 3 and Supplementary Fig. S5), implying ATP conservation through the loss of ABC transporters [21, 22]. In contrast, RD1 specifically possesses DauA, which imports succinate at pH <5 [66]. Therefore, RD1 may have evolved to specifically take up succinate in the acidic rhizosphere soil rather than biosynthesizing it endogenously [21, 22, 67]. As specific genome reduction could be more effective for energy costs than general reduction, RD1 may have evolved as a beneficiary in an energy-saving manner via specific gene loss [10, 24].

Consistent with previous studies on mixed PGPR strains, the combination of RD1 and H3 exhibited better plant protection than the individual treatments (Fig. 4A) [31, 68–71]. Although the mechanisms underlying plant protection by RD1 and H3 remain unknown, there are two possible scenarios. First, syntrophic interaction increases competition with pathogen in the rhizosphere. The combination of RD1 and H3 may improve root colonization more than individual inoculation, owing to improved motility and/or biofilm formation by succinate. Specifically, enhanced biofilm formation may act as a competitive barrier within the root microbiome against pathogens, resulting in the suppression of soil-borne pathogens from invading plant tissues (Supplementary Fig. S6) [63, 72–76]. Second, RD1 and H3 may elicit the induced systemic resistance (ISR) in plants. Similar to other ISR-triggering PGPRs, RD1 and H3 not only reduce disease occurrence but also enhance plant growth without direct antagonism to pathogens (Fig. 4 and Supplementary Fig. S7) [31, 77–79]. In a recent study, two minor strains supported the activity of ISR-triggering key strains for plant protection against *R. pseudosolanacearum* [31], implying that syntrophic interaction between rhizobacteria might play a role in activating ISR in plants.

Our microbiome analysis of viable bacterial colonies suggests that various bacterial taxa in the rhizosphere may live as succinate auxotrophs, which is distinct from conventional nutrient auxotrophy [8–10, 15, 16, 18, 19]. Especially, exogenous succinate affected the viability of bacterial strains in rare taxa, which represented <0.1% abundance, more than the abundant generalists (Fig. 6E). As the importance of rare taxa for plant health has recently been highlighted [80–83], the further studies of energy auxotrophic rare taxa are required to better understand the beneficial plant-microbiota interaction. Furthermore, a computational model has predicted gene loss and auxotrophs in the rhizosphere microbiota [21]. Particularly, root-derived exudates, including organic acids, such as succinate and amino acids, can confer a selective advantage through competition, coexistence, and interdependence in the rhizosphere microbiota [84, 85]. Therefore, we speculate that the effect of the host plant, via root exudate excretion, plays a pivotal role in the syntrophic interactions and inter-kingdom dependency in the complex microbiome [8]. One might expect that succinate in root exudate, or produced from helper microbes, serves as a common good, nourishing the beneficiary bacteria in the rhizosphere. This might, in turn, help to maintain microbial diversity and stabilize a microbial community. There are no reports that exogenous succinate is directly involved in plant defense or growth. However, a robust microbial community stabilized by succinate may activate plant defense or promote plant growth. The ecological significance of succinate in the rhizosphere needs to be further explored.

This study reports the importance of syntrophic interactions between succinate auxotrophic beneficiary and helper bacteria in plant host-associated microbiota. However, our findings could also provide a steppingstone to a better understanding of the unculturability of bacterial species in major bacterial phyla in the rhizosphere and soils [86, 87]. The members of rare taxa are key drivers of the microbiome’s structure and functioning [81, 88, 89]. Based on our results, we propose that, to find new kids on the block in plant-microbiome interaction studies, it is necessary to characterize microbiota-wide auxotrophy and its relevant public goods in complex host-associated communities. We described the evolution of a succinate auxotrophic beneficiary that can proliferate with the aid of helper bacteria in plant-associated rhizospheres. Furthermore, we showed that syntrophic interactions fitting to the BQH play a pivotal role in the promotion of plant growth and health.

## Supplementary Material

Supplementary_information_clean_version_wrae120

## Data Availability

Raw data were deposited in the Sequence Read Archive National Center for Biotechnology Information (SRA-NCBI) under the accession number PRJNA1063572.
