# Supplementary material for: Rhizobacterial syntrophy between a helper and a beneficiary promotes tomato plant health
Source: ISME J. 2024 Jul 2;18(1):wrae120. doi: 10.1093/ismejo/wrae120 (PMC11253211; doi:10.1093/ismejo/wrae120)
Supplement: Supplementary_information_clean_version_wrae120 [file supplementary_information_clean_version_wrae120.docx]

**[Supplementary Information]**

**Rhizobacterial syntrophy between a helper and a beneficiary promotes tomato plant health**

Running title: Syntrophic interaction in plant root microbiota

Authors: Sang-Moo Lee^a,Ϯ^, Roniya Thapa Magar^b,Ϯ^, Min Kyeong Jung^b,Ϯ^, Hyun Gi Kong^b,c^, Ju Yeon Song^d^, Joo Hwan Kwon^b^, Minseo Choi^b^, Hyoung Ju Lee^b^, Seung Yeup Lee^b^, Raees Khan^b,e^, Jihyun F. Kim^d,f^ and Seon-Woo Lee^a,b^*

^a^Institute of Agricultural Life Sciences, Dong-A University, Busan 49315, Republic of Korea

^b^Department of Applied Bioscience, Dong-A University, Busan 49315, Republic of Korea

^c^Department of Plant Medicine, Chungbuk National University, Cheongju 28644, Republic of Korea

^d^Department of Systems Biology and Institute for Life Science and Biotechnology, Yonsei University, Seoul 03722, Republic of Korea

^e^Department of Sciences, National University of Medical Sciences, Rawalpindi, 46000, Pakistan

^f^Microbiome Initiative, Yonsei University, Seoul 03722, Republic of Korea

*Address correspondence to

Seon-Woo Lee,

Department of Applied Bioscience

Dong-A University, Busan 49315

Republic of Korea

E-mail: [seonlee@dau.ac.kr](mailto:seonlee@dau.ac.kr)

^Ϯ^These authors contributed equally to this work.

**[Supplementary Method]**

**Co-inoculation assay for helper and beneficiary bacteria**

To investigate the effect of proximity between RD1 and H3, 5-μL of cell suspension was co-inoculated in a diagonal row on R2A medium creating a V-shape of increasingly closer inoculation sites[1]. For physical separation between the RD1 and H3 inoculants, cell suspensions were inoculated on R2A medium in an I-plate (SPL Lifesciences Co., Seoul, South Korea) and incubated at 30℃.

**Assessment of various substrates for RD1 induction**

To test the nutrient and growth media preferences of beneficiary RD1, various medium contained different factors at specific concentrations were used to culture RD1 cell (Supplementary Table 1). These included both agar medium and liquid medium of the following laboratory standard media: King’s B medium (20 g/L proteose peptone No. 3, 10 ml/L glycerol, 1.5 g/L K_2_HPO_4_, and 1.5 g/L MgSO_4_), nutrient agar medium (NA, Becton Dickinson, MD, USA), CPG agar medium, tryptic soy agar medium (TSA, Becton Dickinson, MD, USA). For heat killed bacterial cells, the cell suspension of the H3 strain or the mixture of H3 and RD1 cells were autoclaved at 121℃ for 15 min. For soil agar medium, forest and upland soils were used and RD1 was spread over the media followed by incubation at 25, 30, and 37°C. Media detoxification was carried out by growing helper H3 cells for 2 to 25 days and spreading RD1 after the subsequent removal of the H3 colony. To utilize a cellulose membrane filter, RD1 was spread on agar medium, and H3 was incubated onto the cellulose membrane filter (0.2 and 0.45 μm in pore size diameter, ADVANTEC Co., Japan) placed on the medium at 30°C for 2 weeks to examine RD1 colony formation.

**Recombinant DNA technology and transposon mutagenesis of H3**

Plasmid preparation, restriction endonuclease digestion, DNA ligation, and other recombinant DNA techniques were performed according standard protocol[2]. To generate helper H3 mutants, transposon was randomly inserted into the H3 genome by triparental mating, as described previously with minor modification[3]. The *E. coli* donor with the pTn*Mod*-Okm plasmid and the *E. coli* HB101 helper with the plasmid pRK2013 were used. Transconjugants carrying transposon insertions were selected on mannitol-glutamate (MG) agar medium supplemented with kanamycin.

To identify genes involved in helper function, a total of 5,000 helper H3 mutants were co-cultured with RD1 in R2A medium and assessed by incubating at 25°C for 2 weeks. The gDNA fragment of mutant H3-808 carrying a transposon was subcloned into pUC119 to identify the location of the transposon-insertion site, as previously described with slight modification[3]. The transformed *E. coli* carrying the plasmid with transposon-inserted gDNA fragments were selected on LB agar medium containing kanamycin and ampicillin. Recombinant plasmid DNA was sequenced at Macrogen Inc. (Seoul, South Korea). Open reading frames (ORFs) of the transposon-inserted site were analyzed using CL Genomics and BLASTx (Supplementary Table 2).

**Investigation of the effect of the substance related to TCA cycle and GABA shunt pathway for RD1 cultivation**

To validate the specificity of succinate in RD1 growth, RD1 was cultivated in R2A medium containing 1.0% citric acid (Junsei Chemical Co., Tokyo, Japan), α-ketoglutaric acid (Sigma-Aldrich, MA, USA), malic acid (Sigma-Aldrich, MA, USA), L-glutamic acid (Sigma-Aldrich, MA, USA), γ-aminobutric acid (Sigma-Aldrich, MA, USA) adjusted pH 7.2 for 3 days at 30℃.

**Investigation of the correlation between succinate and nitrogen resources in RD1 growth.**

To check the dependency of succinate on RD1, RD1 was cultivated in a water agar medium with serially diluted R2B broth (1, 1/10, and 1/100). To find R2A component synergizing succinate for RD1, RD1 was inoculated on minimal medium (0.3-g/L K_2_HPO_4_ and 0.05-g/L MgSO_4_) containing individual nutrient sources constituting R2A medium and 2% succinate.

**Preparation of bacterial suspension for *in planta* experiments**

Helper bacterium H3 was cultured in R2A agar medium for 48 h at 30*°*C. Beneficiary bacterium RD1 was co-cultured with H3 bacteria (H3 suspension spotted in the middle of RD1 bacterial lawn) in R2A at 30*°*C until RD1 could form bacterial colonies. Bacterial cells of H3 and RD1 on R2A medium were suspended separately with SDW and adjusted to an OD_600_ of 1.0. For the mixture of H3 and RD1, equal amounts of each H3 and RD1 suspension with OD_600_ = 1.0 were mixed. *R. pseudosolanacearum* strain SL341 was cultured in CPG agar medium (1 g/L casamino acid, 10 g/L peptone, and 5 g/L glucose), supplemented with 0.005% (w/v) 2,3,5-triphenyl tetrazolium chloride (TZC), at 30*°*C for 48 h. *R. pseudosolanacearum* SL341 bacterial suspension for inoculation to plants was prepared in SDW and adjusted to OD_600_ = 0.2 (2 × 10^8^ CFU/mL).

**The measurement of *Ralstonia pseudosolanacearum* SL341 in tomato stem.**

To calculate the population of SL341 in the stems of tomato plants, the suspension of SL341 were inoculated using the soil-soaking method, and stem samples were collected at 9 dpi. After vigorous vortexing, stems were incubated in sterile water for 10 min. Subsequently, a series of diluted samples were plated on SMSA medium[4] to determine the colony forming units (cfu) per gram of stem fresh weight.

**Assesement of antagonistic activity against *Ralstonia pseudosolanacearum* SL341 *in vitro*.**

To examine the antagonistic activity of the RD1 and H3 mixture against *R. pseudosolanacearum* SL341, lawn of *R. pseudosolanacearum* (OD_600_ = 0.1) was prepared by mixing 250-μL of bacterial suspension with R2A soft agar (R2A with 0.6% agar). Then, the mixture of RD1 and H3 (10-μL; OD_600_ = 0.1) were dropped in the middle of the lawn of *R. pseudosolanacearum*. Controls were inoculated with kanamycin (0.5-mg/mL; positive control) or SDW (negative control). The inhibition zone was observed after 2 days incubation at 30*°*C.

**Microtiter plate biofilm production assay and swimming motility test**

Biofilm production by RD1 and H3 was quantified by crystal violet staining, as described previously with slight modification[5]. Briefly, each bacterial strain was suspended in R2B broth medium (OD_600_ = 1.0) with or without 1% succinate. For the RD1+H3 mixtures, RD1 and H3 bacterial suspensions (OD_600_ = 1.0) were mixed with an equal volume. R2B broth was used as a negative control. 100-μL each suspension was transferred to 96-well polyvinylchloride (PVC) microplate (Corning, NY, USA). Parafilm-covered microplate was incubated for 24 h at 30℃. Incubated suspension was stained with 25-μL of 0.1% crystal violet for 15 min at 30℃. Subsequently, planktonic cells were removed, and the plates were washed three times with SDW and air dried. The cell bound crystal violet was dissolved in 200-μL ethanol. Finally, biofilm production was quantified by measuring the optical density (OD_600_) using a Multiskan^TM^ GO microplate spectrophotometer (Thermo Fisher Scientific).

Swimming motility was performed in R2A medium containing 0.3% agar. Next, 10-μL of bacterial suspension (OD_600_ =1.0) was spotted on R2A medium with or without 1% succinate. For the co-inoculation of RD1 and H3, cell suspensions of RD1 and H3 were inoculated at a distance of 3-cm on R2A medium containing 0.3% agar. The agar medium was incubated at 30℃ and the swimming motility of each strains was observed at 3 dpi.

**Microbiome analysis pipelines**

Demultiplexing and quality filtering were performed on raw FASTQ sequences of the 16S rRNA amplicons. De-noising, de-replication, and chimera filtering of the sequence data were conducted using DADA2[6] and clustered into groups sharing 99% identity within the VSEARCH[7]. The dataset was filtered using the QIIME "taxa filter-table" command to remove singletons and nonbacterial species, including archaea, mitochondria, chloroplasts, and Cyanobacteria. To assign taxonomy to clustered the Operational Taxonomic Units (OTUs), a Naïve Bayes classifier was trained on the SILVA (v. 138.1) database using the classify-sklearn command within the feature-classifier plug-in[8]. A phylogenetic tree was generated based on the 16S rRNA gene fragments using MAFFT-aligned representative OTU sequences in FastTree, with the tree rooted at the midpoint. This phylogeny was used for both the alpha and beta diversity analyses[9].

Bacterial alpha and beta diversity metrics were used to compare the viable bacterial community composition between different treatments using a rarefied OTU table. To standardize the depth of the reads after subsampling, the rarefy_even_depth function of the PhyloSeq package was used to generate the OTU table. The alpha and beta diversities were plotted by importing the required normalized OTU table and phylogeny using PhyloSeq in R (version 4.1.3) and graphed using ggplot2[10,11]. The bacterial alpha diversity across succinate treatment and non-treatment groups was calculated using two metrics (observed OTUs and evenness). Principal coordinate analysis was performed using weighted UniFrac distance for inter-sample analysis. Relative abundance analysis was performed by normalizing the OTUs and expressing the taxonomic composition of each sample as the ratio of individual OTU counts to the total counts of all OTUs within that sample. Additionally, random forest[12] was performed using the R program to select important bacteria responding to succinate treatment using the OTU table obtained from the microbiome analysis.

1. Berendsen, R.L. et al. Disease-induced assemblage of a plant-beneficial bacterial consortium. *ISME J*. **12**, 1496-1507 (2018).

2. Sambrook, J., Fritsch, E.F. & Maniatis, T. (eds). Molecular colning, A laboratory manual. (CSHL Press, 1989).

3. Choi, K. et al. Contribution of the *murI* gene encoding glutamate racemase in the motility and virulence of *Ralstonia solanacearum*. *Plant Pathol. J*. **36**, 355 (2020).

4. Schaad, N.W., Jones, J.B. & Chun, W. (eds). Laboratory guide for the identification of plant pathogenic bacteria. (APS Press, 2001).

5. O'Toole, G.A. & Kolter, R. Initiation of biofilm formation in *Pseudomonas fluorescens* WCS365 proceeds via multiple, convergent signalling pathways: a genetic analysis. *Mol. Microbiol*. **28**, 449-461 (1998).

6. Callahan, B.J. et al. DADA2: High-resolution sample inference from Illumina amplicon data. *Nat. Methods* **13**, 581-583 (2016).

7. Rognes, T., Flouri, T., Nichols, B., Quince, C. & Mahé, F. VSEARCH: a versatile open source tool for metagenomics. *PeerJ* **4**, e2584 (2016).

8. Bokulich, N.A. et al. Optimizing taxonomic classification of marker-gene amplicon sequences with QIIME 2’s q2-feature-classifier plugin. *Microbiome* **6**, 1-17 (2018).

9. Price, M.N., Dehal, P.S. & Arkin, A.P. FastTree 2-approximately maximum-likelihood trees for large alignments. *PLoS One* **5**, e9490 (2010).

10. McMurdie, P.J. & Holmes, S. phyloseq: an R package for reproducible interactive analysis and graphics of microbiome census data. *PLoS One* **8**, e61217 (2013).

11. Wickham, H. Programming with ggplot2. *in ggplot2: Elegant graphics for data analysis* (eds Wickham, H.) 241-253 (Springer, 2016).

12. Liaw, A. & Wiener, M. Classification and regression by randomForest. *R news* **2**, 18-22 (2002).


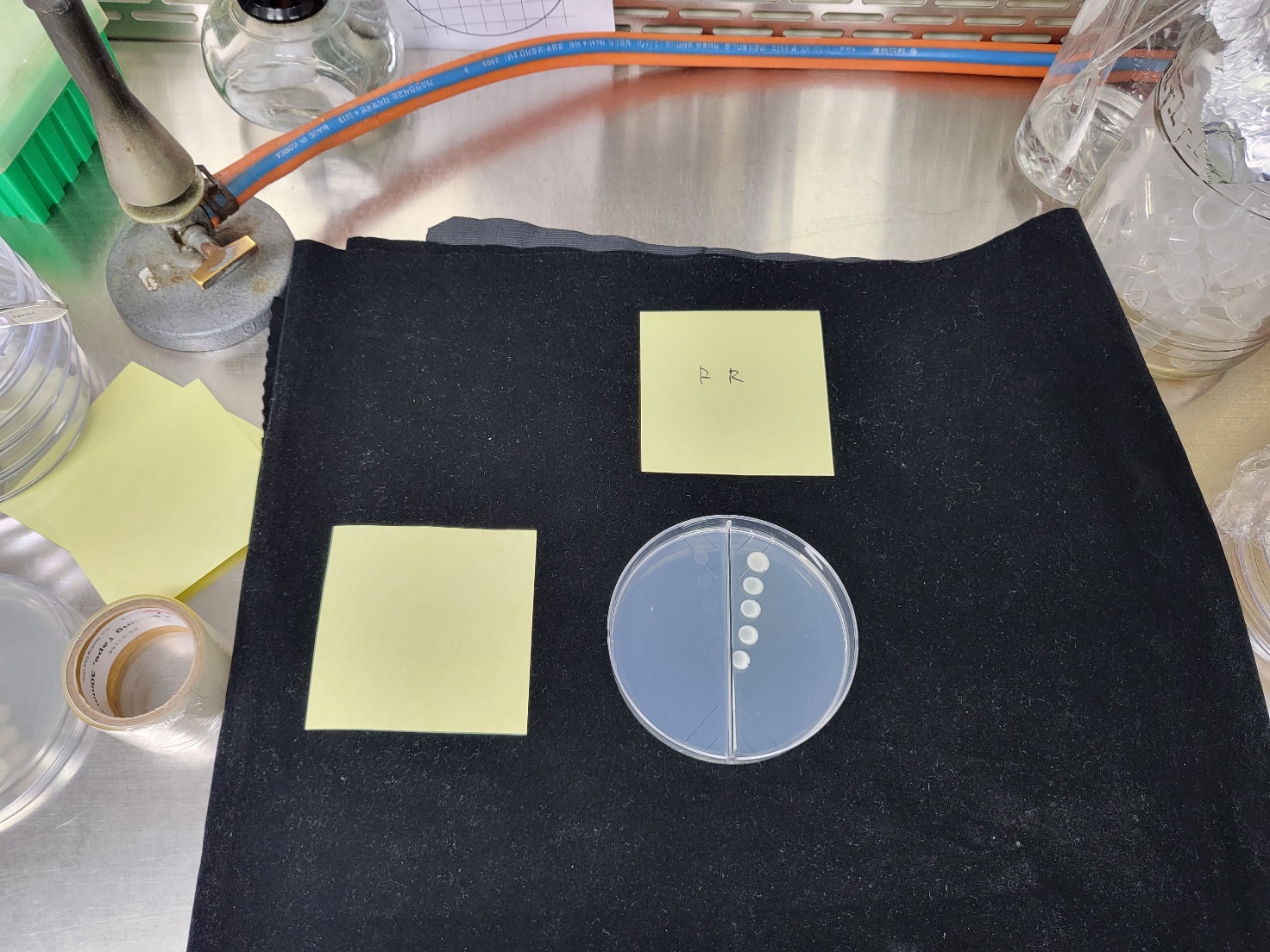


RD1

H3


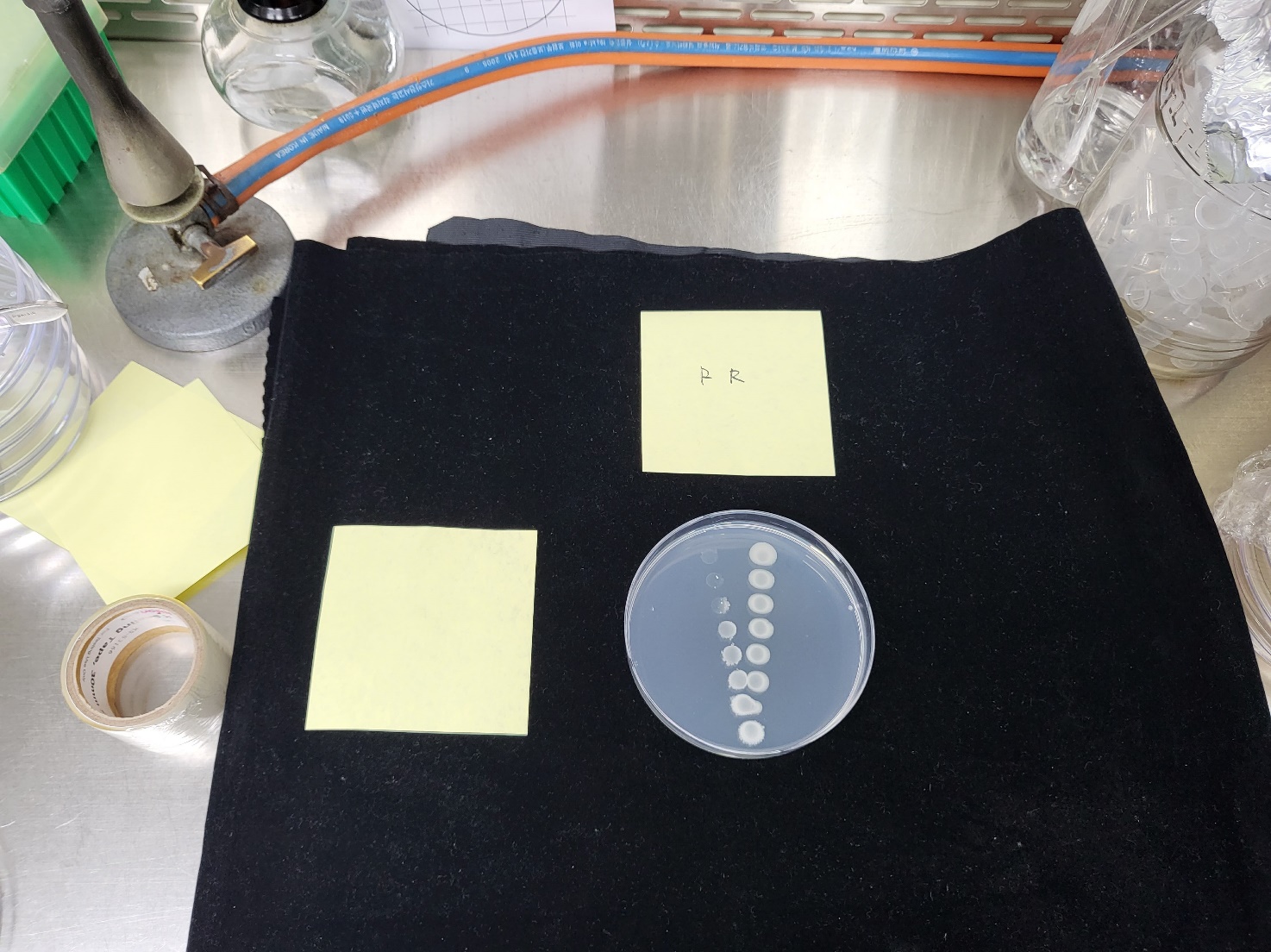


RD1

H3


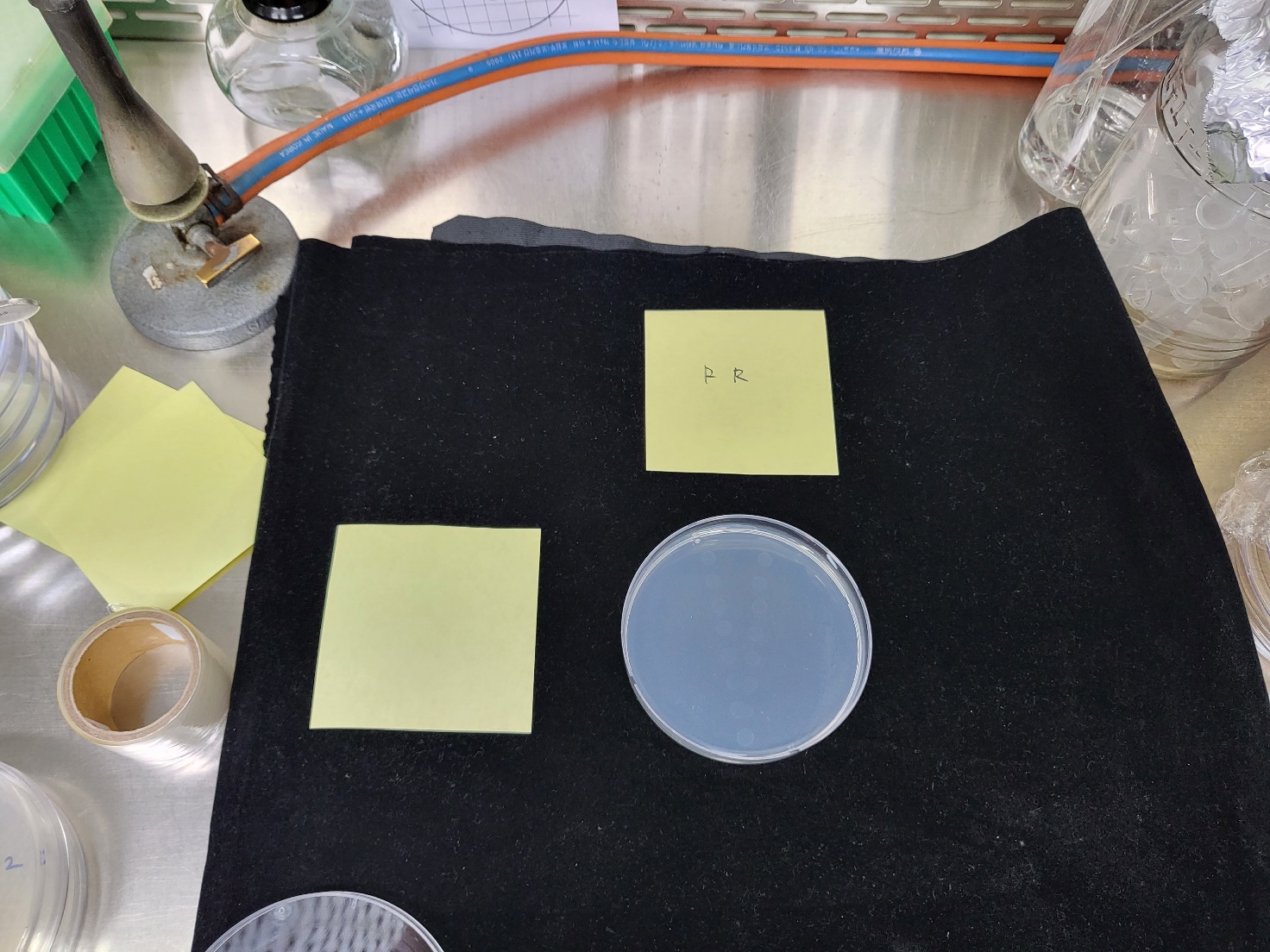


RD1

RD1


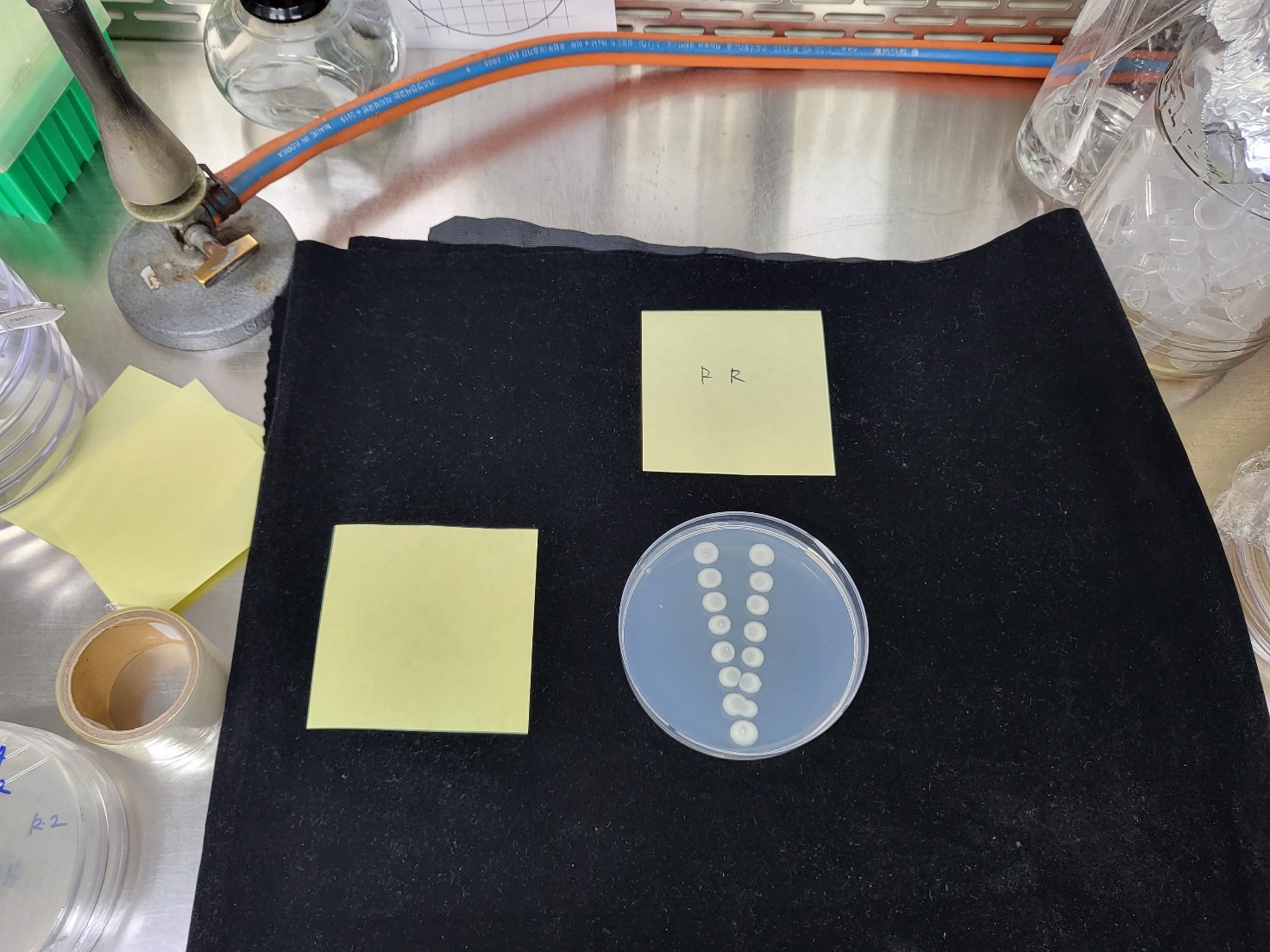


H3

H3

**Supplementary Fig. S1 The growth of RD1 depends on the H3 on R2A medium**. In V-shape assay system, the growth of RD1 was determined by the proximity of H3; however, when the diffusion of H3-derived compound was blocked, the growth of beneficiary RD1 were reduced in I-plate system.

**Succinate**

α-ketoglutarate

L-Glutamate

GABA

**GABA-S**

**TCA cycle**

SSA

Malate

Citrate

**(A)**

**(B)**


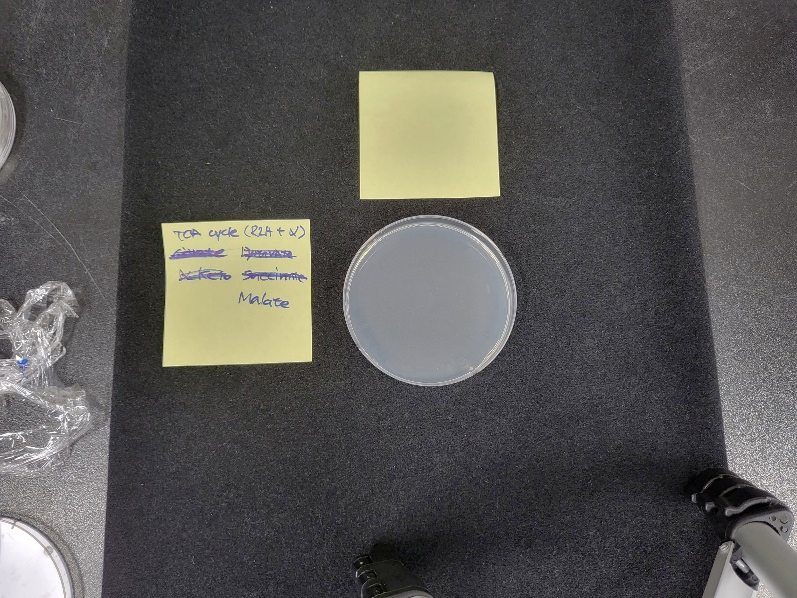


**Malate**


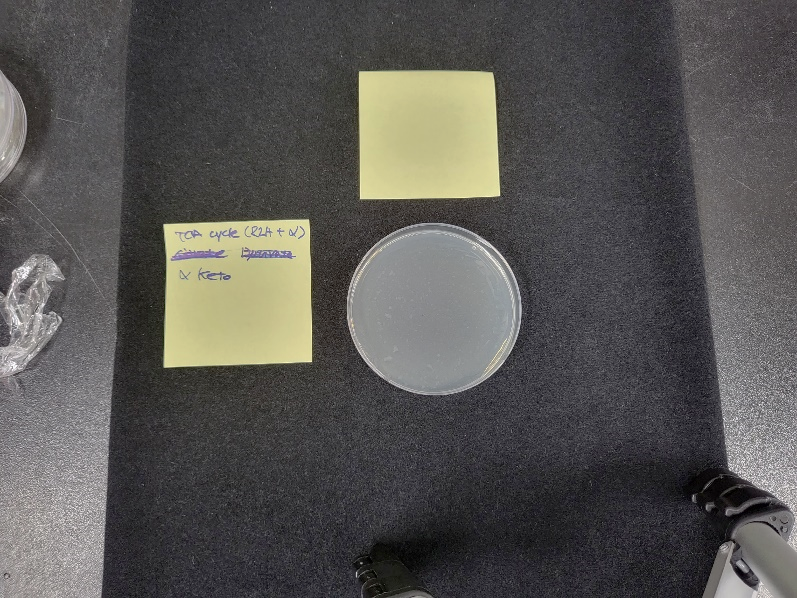


**α-ketoglutarate**


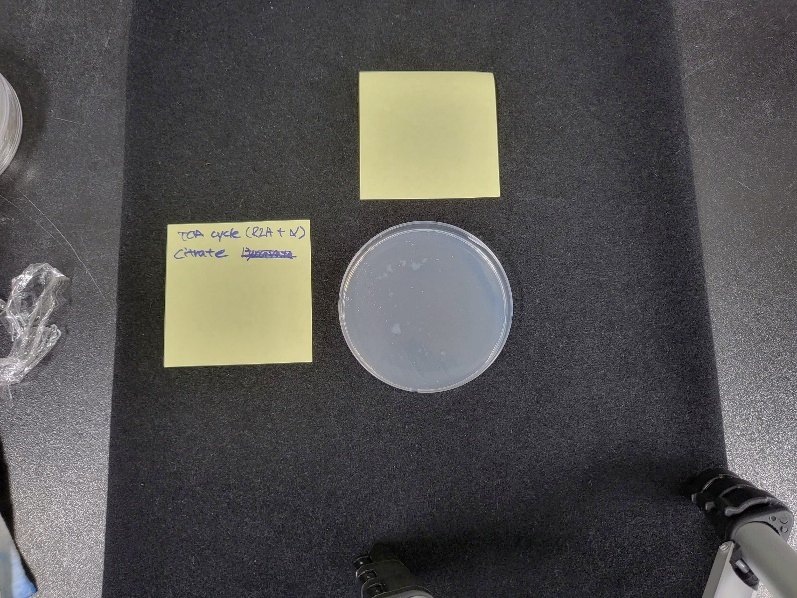


**Citrate**


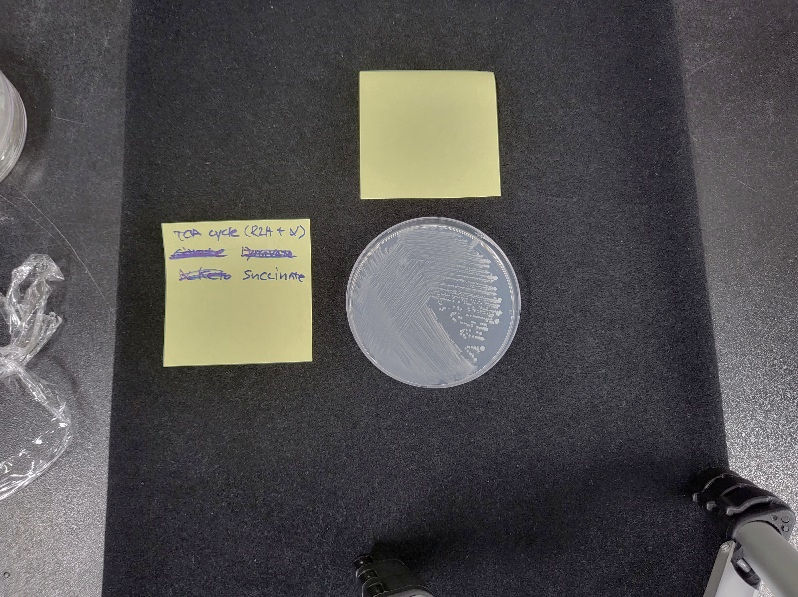


**Succinate**


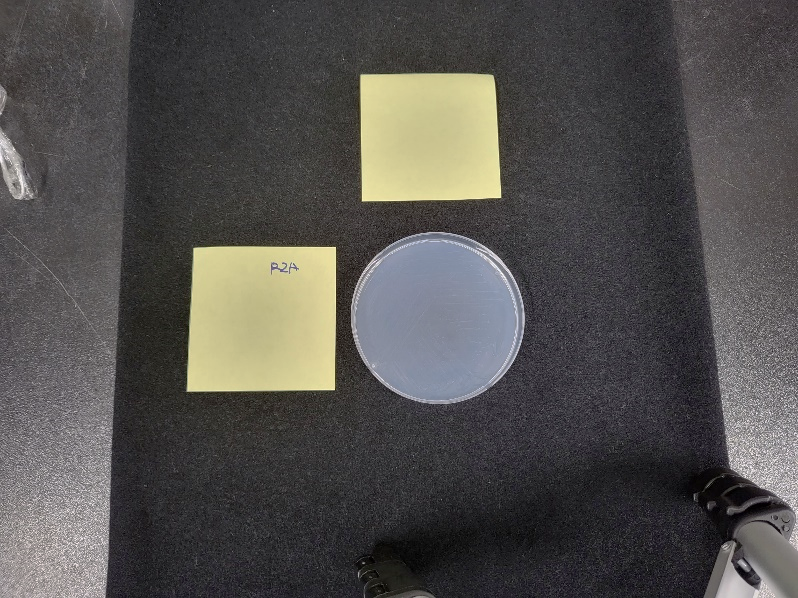


**Control**

**(C)**


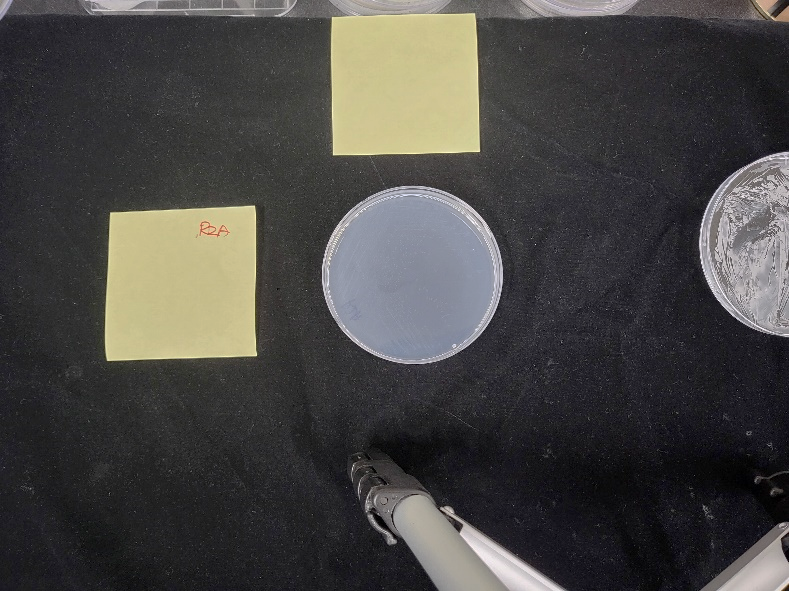


**Control**


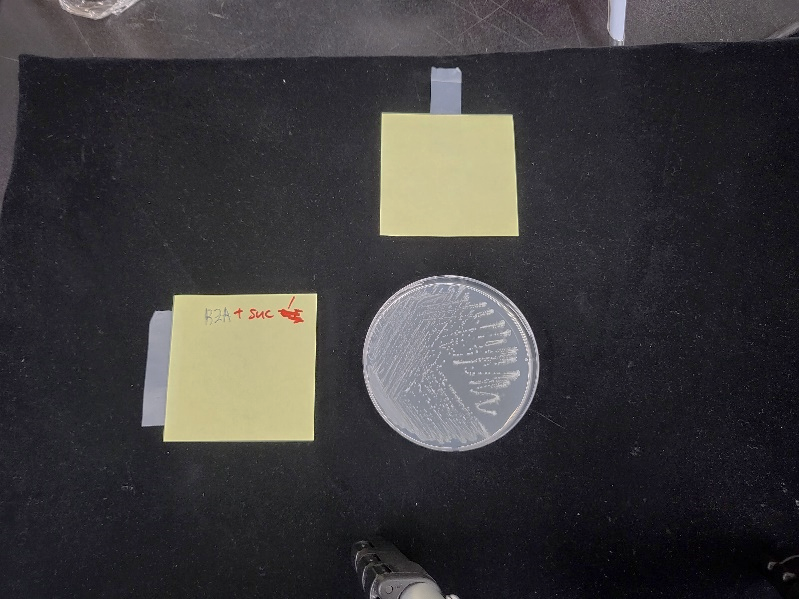


**Succinate**

**GABA**


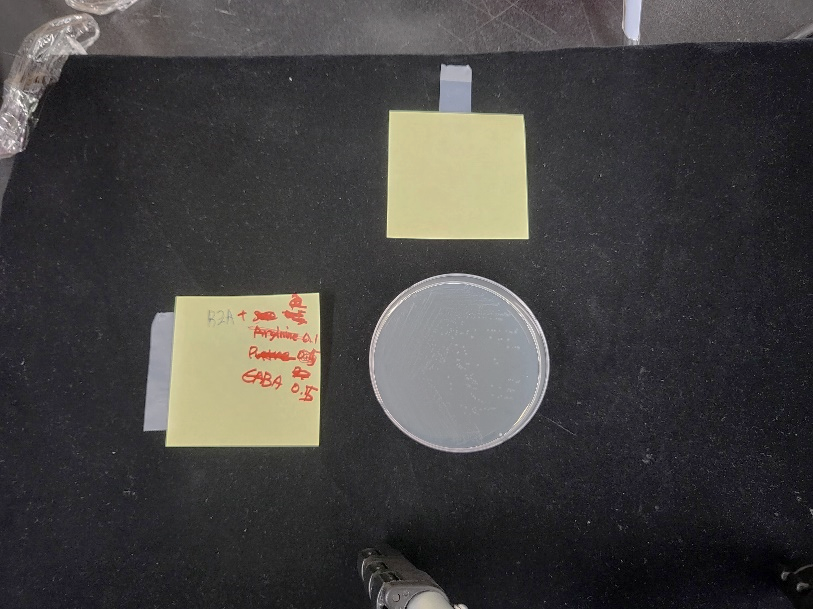


**L-glutamate**


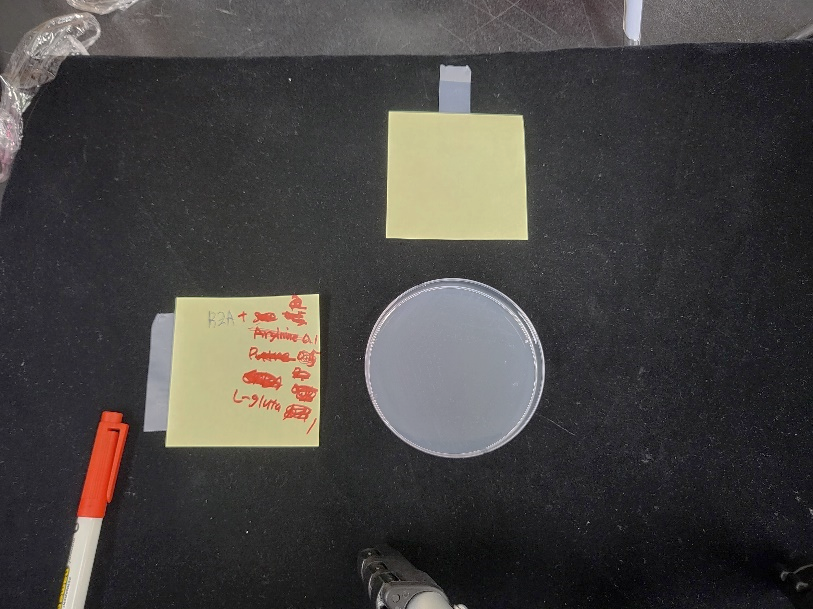


**Supplementary Fig. S2. The effect of TCA cycle and GABA shunt intermediates on growth of RD1.** (A) Tested intermediates involved in TCA cycle and GABA shunt pathway. Blue colored intermediates were used for RD1 growth. (B) RD1 was inoculated in R2A agar medium with 1 % intermediates of TCA cycle (citrate, α-ketoglutarate, and malate) and (C) GABA shunt (γ-aminobutyric acid and L-glutamate). This picture was taken at 5 days after post inoculation of RD1. GABA-S, GABA shunt pathway; SSA, Succinic semialdehyde.


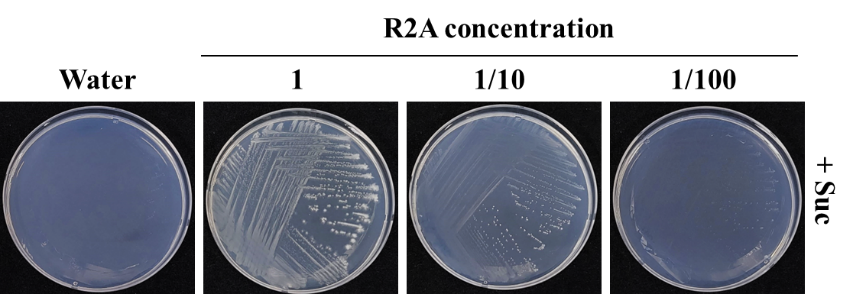


**(A)**

**(B)**


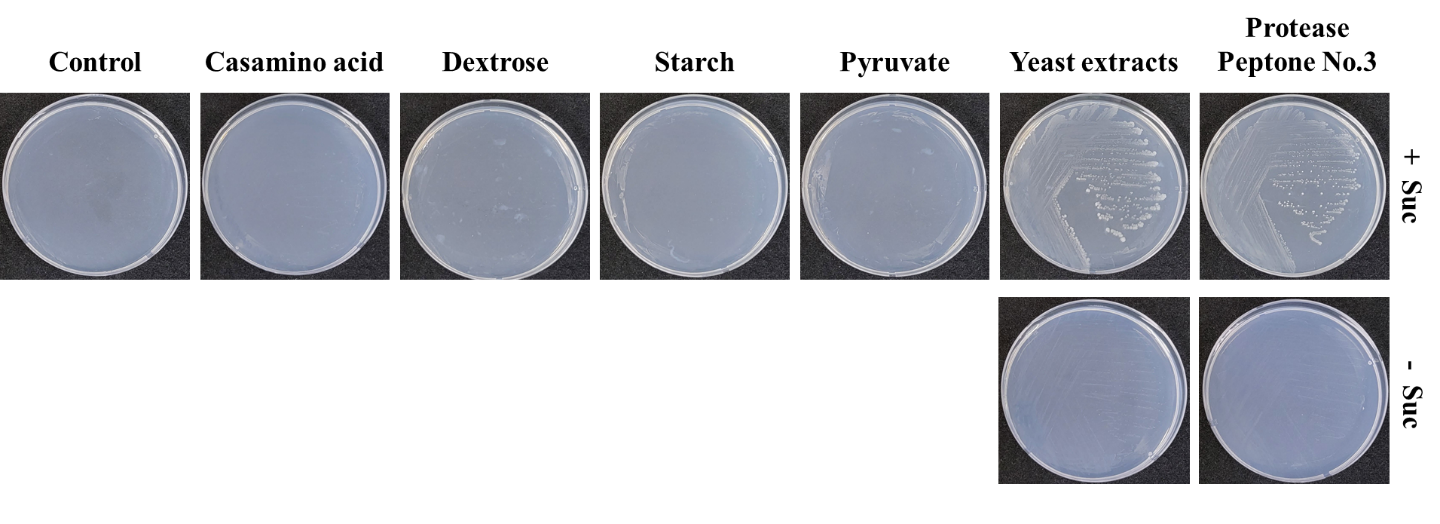


**Supplementary Fig. S3. Combination of succinate and nitrogen source enhances RD1 growth.** (A) Succinate enhanced RD1 growth with R2A component(s). Water, water agar medium without R2A; 1, 1/10, and 1/100, dilution factor of R2A concentration in agar medium (B) RD1 cell suspension was inoculated on minimal agar medium (0.3 g/L K_2_HPO_4_ and 0.05 g/L MgSO_₄_) containing individual nutrient sources constituting R2A medium and 2 % succinate. Control, minimal agar medium; casamino acid, minimal agar medium containing 0.5 g/L casamino acid; Dextrose, minimal agar medium containing 0.5 g/L dextrose; Starch, minimal agar medium containing 0.5 g/L starch; Pyruvate, minimal agar medium containing 0.3 g/L pyruvate, Yeast extracts, minimal agar medium containing 0.5 g/L yeast extracts, Protease Peptone No. 3, 0.5 g/L protease peptone No. 3; + Suc, with 2 % succinate; - Suc, without succinate

**
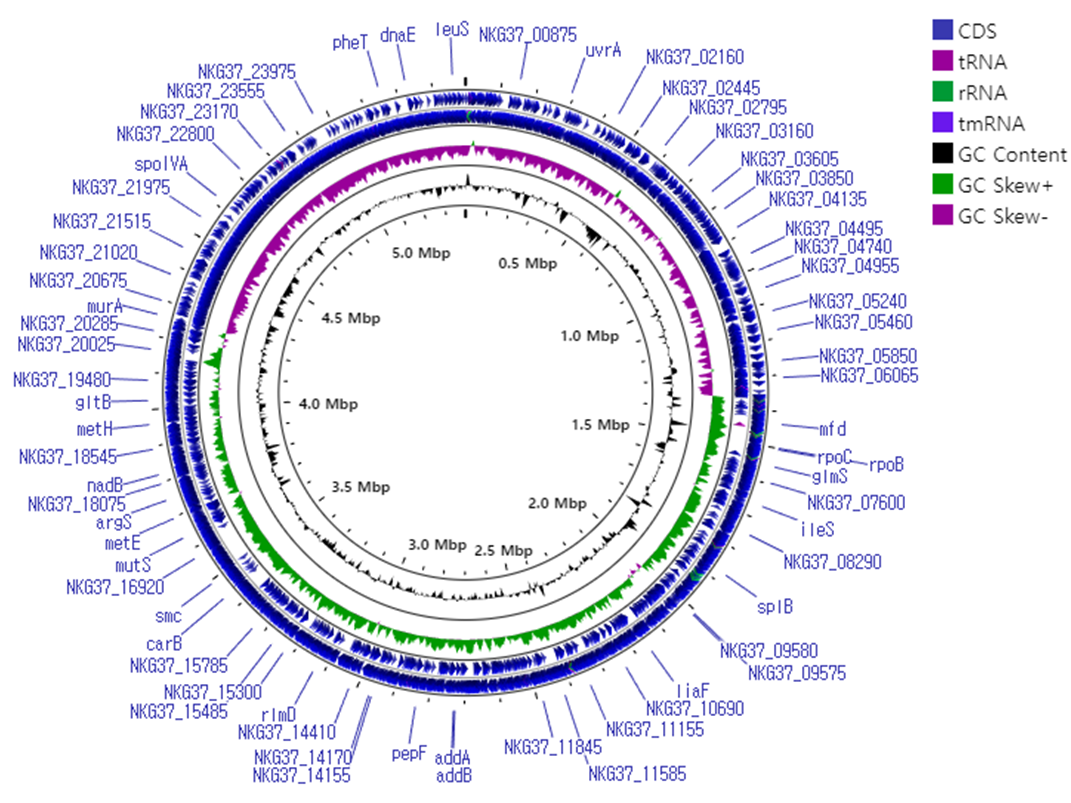
**

**Supplementary Fig. S4. Schematic of the complete RD1 genome.** Circles represent the following features labeled from the outside to inside. From the outside, the first and second circles represent positive and negative strand genes, respectively. Blue, purple, green, and cyan arrows indicate CDS, tRNA, rRNA, and tmRNA, respectively. The third circle shows the GC skew (GC skew= [G $-$ C]/[G + C]; green means greater than 0, purple means less than 0). The fourth circle represents the GC content. The innermost circle indicates the scale in bp. Proksee (https://proksee.ca/) software was used to draw the genomic circle map.


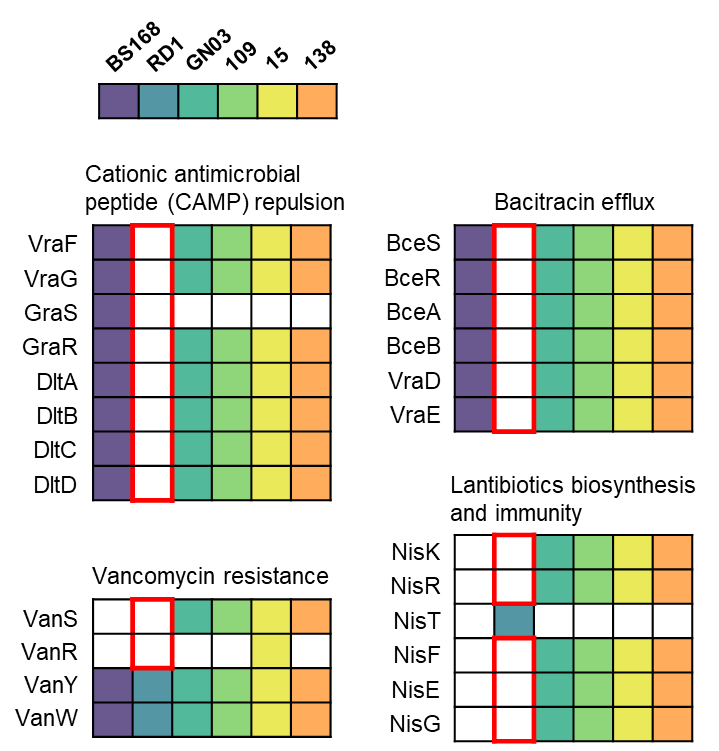


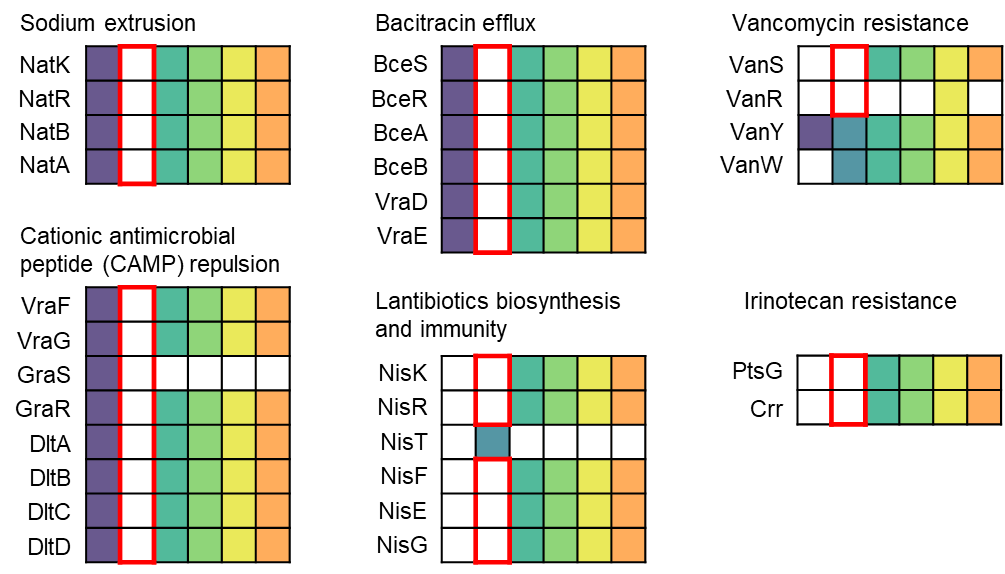


**Supplementary Fig. S5. Loss of gene involved in ABC transporters for antibiotics efflux in RD1.** Red box: A gene lacking in RD1, BS168, *Bacillus subtilis* strain 168; RD1, *Niallia* sp. RD1; GN03, *Niallia circulans* GN03; 109, *N. circulans* strain PK3_109; 15, *N. circulans* strain PK3_15; 138, *N. circulans* strain PK3_138.

**Supplementary Fig. S6.** **The population of *R. pseudosolanacearum* SL341 in stem of Zuiken tomato.** Bacterial cell count of SL341 in Zuiken tomato stems at 9 days post inoculation. H3, RD1, and RD1+H3 are treatment of H3 alone, RD1 alone and mixture of RD1 and H3, respectively. Negative control (Control) was treated with sterile distilled water. Different letters on the bar represent significant difference among means of 5 plants by Dunnett's test.


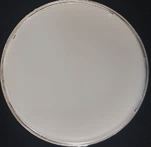

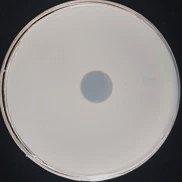

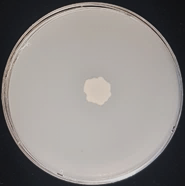


BA

CC

A

**Supplementary Fig. S7.** **Antagonistic activity of mixture of H3 and RD1 against *R. pseudosolanacearum*.** Co-culture of the mixture of helper H3 and beneficiary RD1 bacteria with pathogen *R. pseudosolanacearum* SL341 strain on R2A agar medium. 10μL of (A) H3 and RD1 mixture (OD_600nm_ = 0.1 each), (B) kanamycin (0.5 mg/mL; positive control), or (C) sterile distilled water (negative control) were dropped on a lawn of *R. pseudosolanacearum*, and results were observed after 2 days.

**(B)**

**(A)**


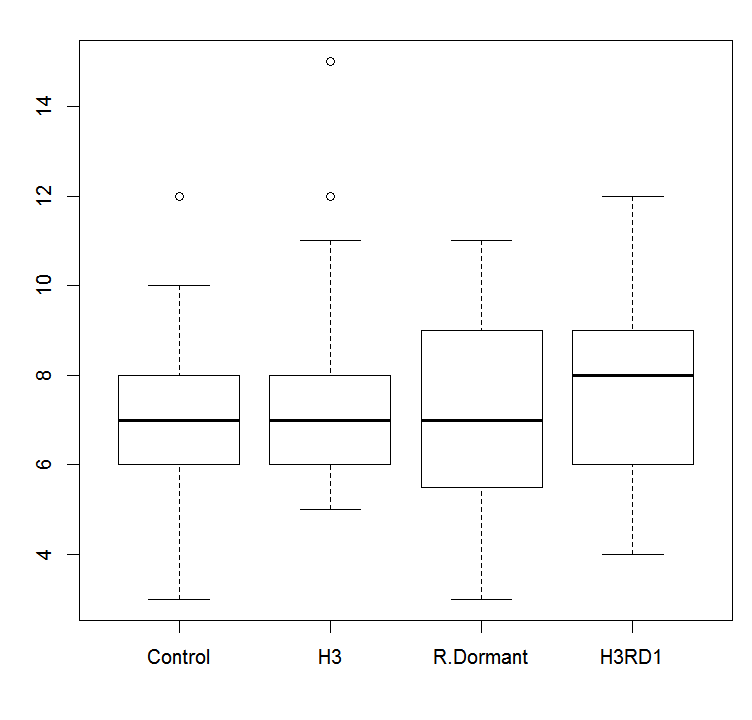


Root fresh weight (mg)

a

a

a

a


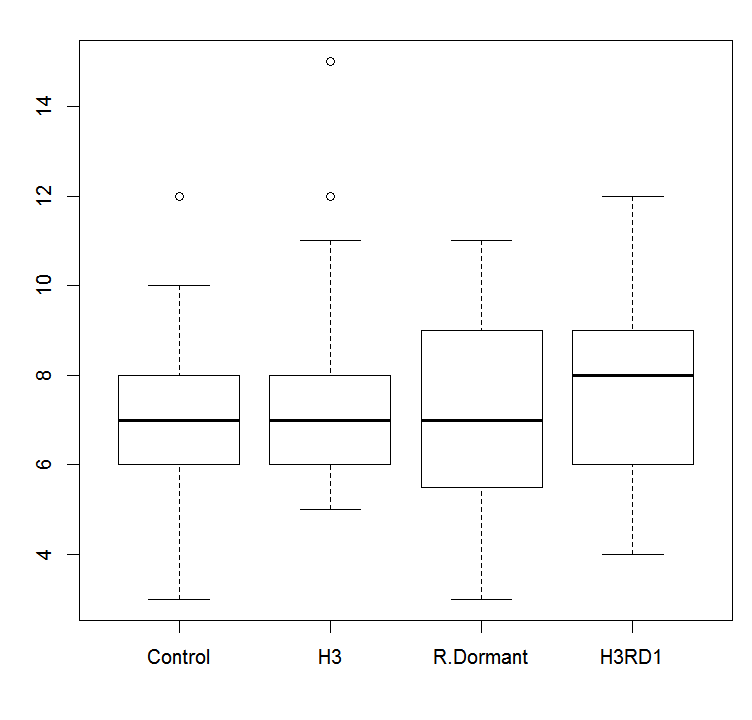


a

a

Control

RD1+H3

RD1

H3


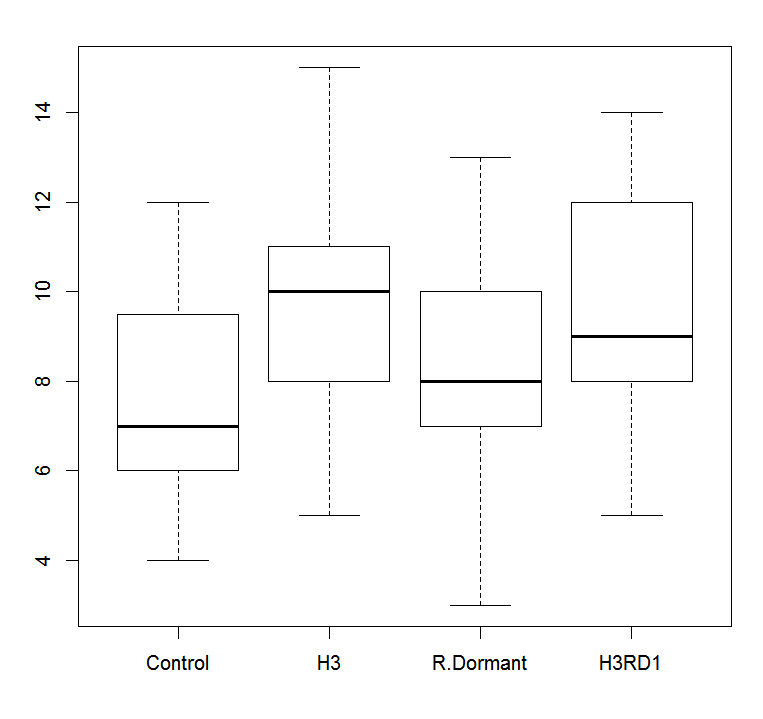


Shoot fresh weight (mg)

b

a


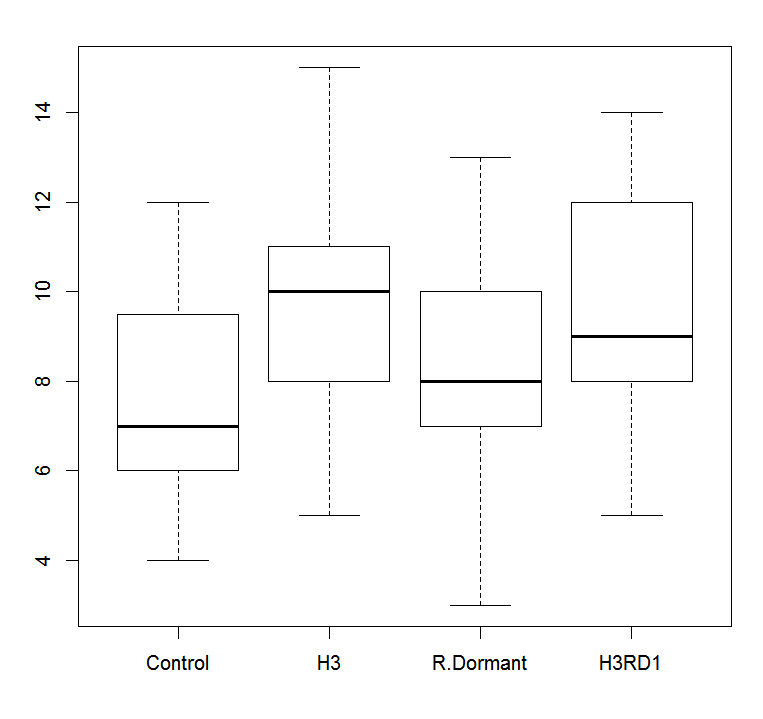


a

ab

Control

RD1+H3

RD1

H3

a

**Supplementary Fig. S8.** **Tomato seedling growth promotion with bacterial treatment.** Fresh weight of (A) hypocotyl and (B) root were measured 5 days after treatment. H3, R D1, and RD1+H3 are treatment of H3 alone, RD1 alone and mixture of RD1 and H3, respectively. Negative control (Control) was treated with sterile distilled water. Different letters on the bar represent significant difference among means of 30 seedlings by Tukey’s multiple range test. **Supplementary Fig. S9. Design of specific primers for the measuring the population of H3 and RD1 in rhizosphere soil.**


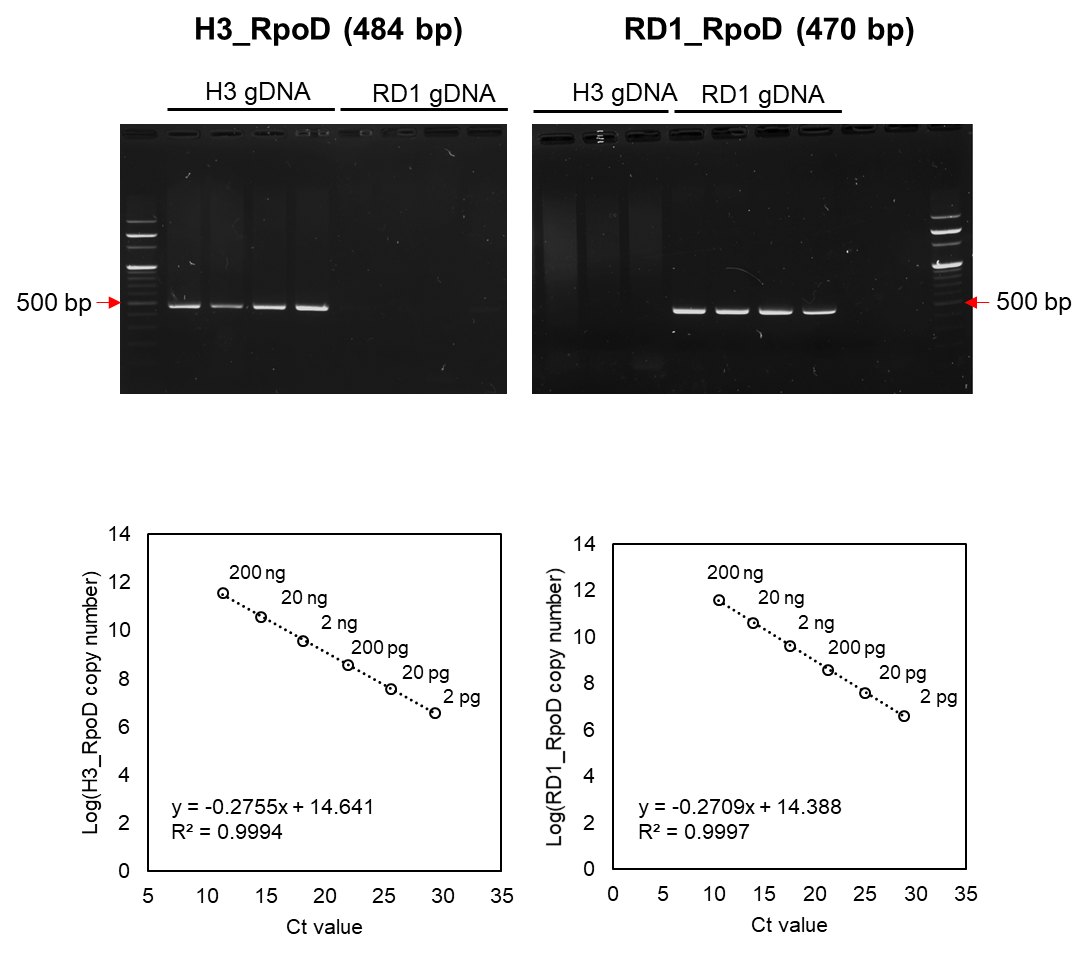


**H3 *rpoD***

**RD1 *rpoD***

**(C)**

**(A)**


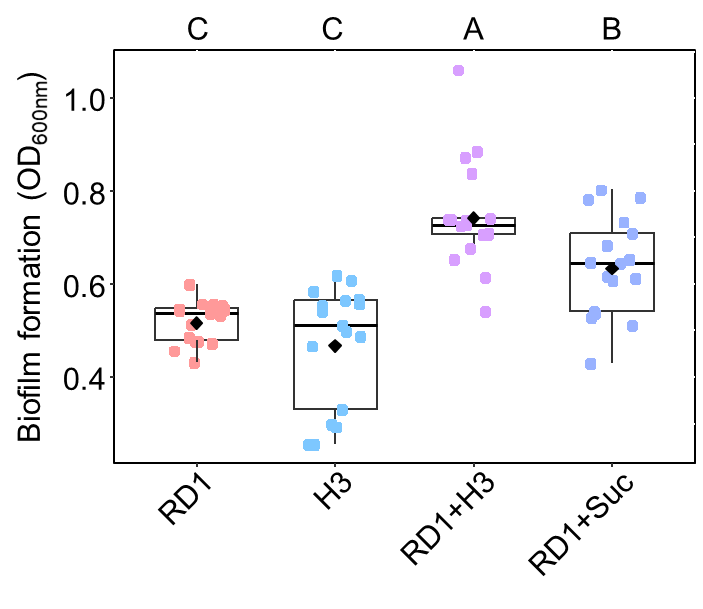

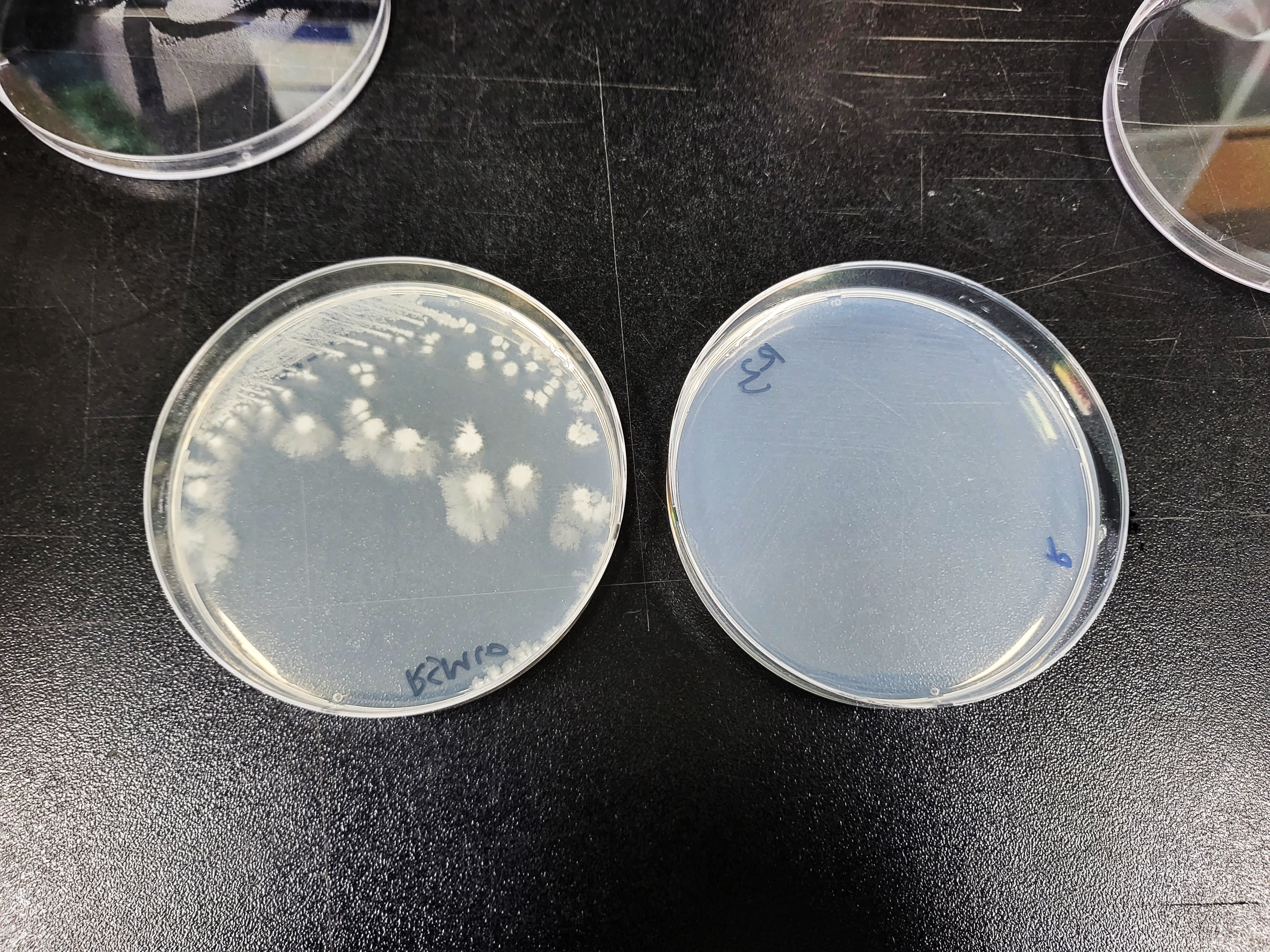


- Suc

+ Suc

**(B)**


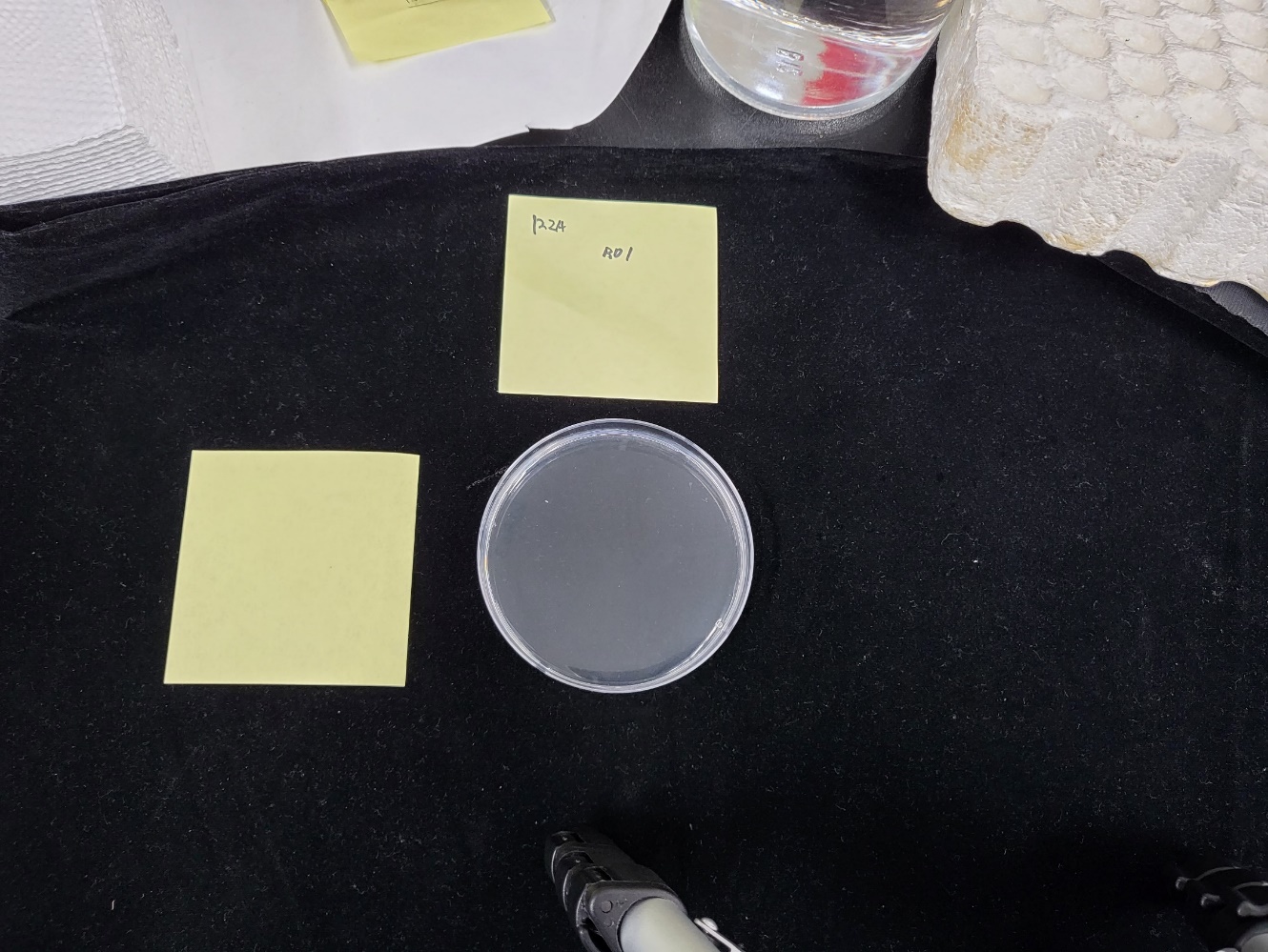

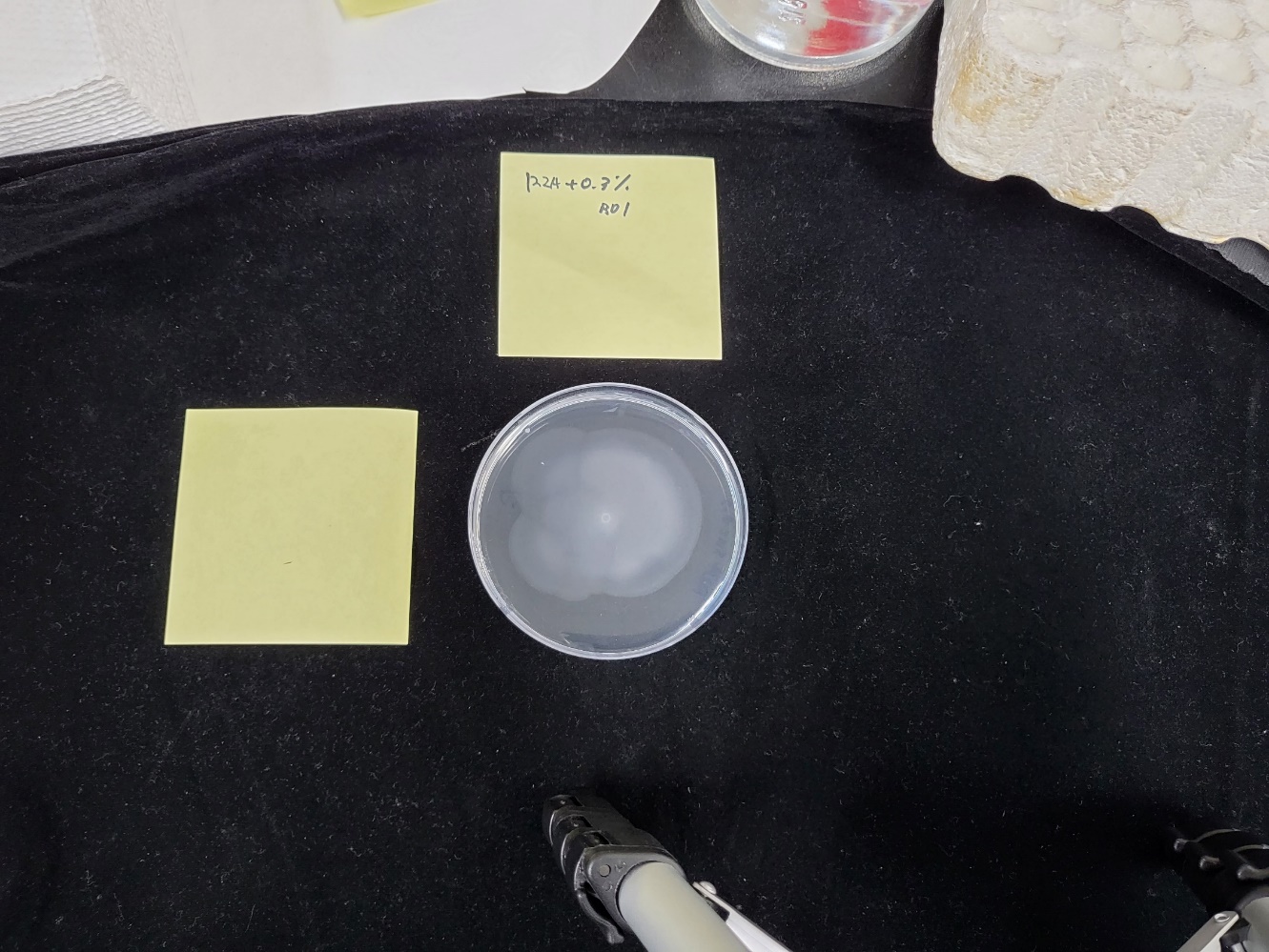


- Suc

+ Suc

**Supplementary Fig. S10. Exogenous succinate enhances the biofilm formation and cell motility of RD1 without helper.** (A) 1% succinate treatment promoted the cell motility of RD1 without helper on R2A agar medium. This pictures were taken at 7 dpi. (B) Swimming motility of single RD1 was promoted by 1% succinate treatment under low agar condition. – Suc, No succinate treatment; + Suc, 1% succinate treatment. (C) Exogenous succinate enhanced the biofilm formation of RD1 without helper. Biofilms were quantified as absorbance at 600 nm (OD_600_) following crystal-violet staining method using 96-well PVC microplate. RD1, single RD1 culture solution; H3, single H3 culture solution; RD1+H3, mixture of RD1 and H3; RD1+Suc, RD1 culture solution with 1% succinate.


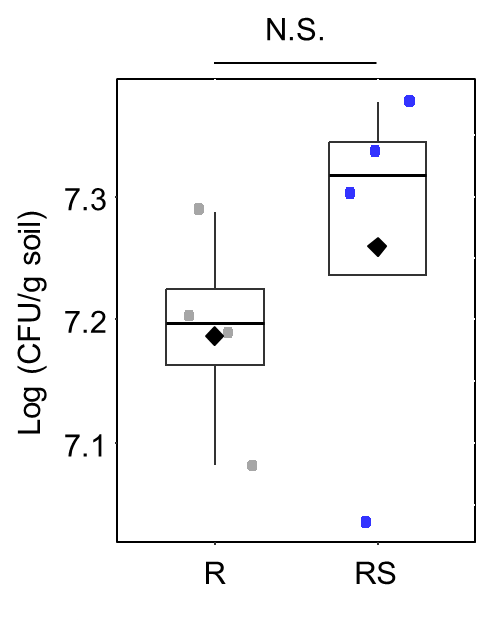


**Supplementary Fig. S11. The total bacterial population of native soil in R2A containing succinate.** R, R2A agar medium; RS, R2A agar medium with 1% succinate. (Student’s T-test, **P* < 0.05, ***P* < 0.01, ****P* < 0.001, N.S., Not significant).

**Supplementary Fig. S12. Rarefaction curves for microbial community comparison of total viable colonies grown at R2A agar medium containing succinate by 16S rRNA amplicon sequencing.** R, R2A agar medium; RS, R2A agar medium with 1% succinate.


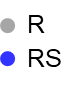

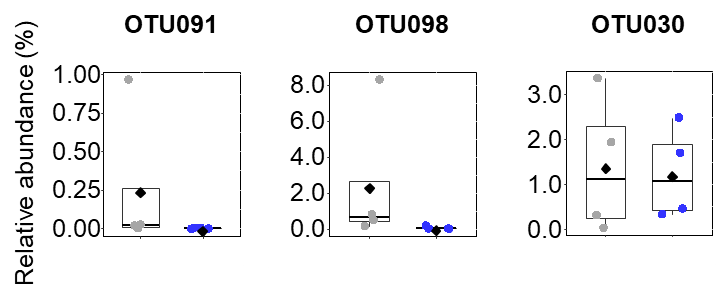


**Supplementary Fig. S13. Relative abundance of OTUs which were not enriched by exogenous succinate.** R, R2A agar medium; RS, R2A agar medium with 1% succinate.

**Supplementary Table S1. Genome information of *Niallia* sp. RD1, *Bacillus subtilis* 168, and four plant-associated *Niallia* species.**

|  | ***Bacillus subtilis* 168** | ***Niallia* sp. RD1** | ***N. circulans* GN03**^*^ | ***N. circulans* PK3_109** | ***N. circulans* PK3_15** | ***N. circulans* PK3_138** |
| --- | --- | --- | --- | --- | --- | --- |
| Accession | NC_000964.3 | NZ_CP100420.1 | NZ_CP053315.1  NZ_CP053316.1 | NZ_CP026031.1 | NZ_CP026040.1 | NZ_CP026033.1 |
| Size | 4,215,606 | 5,394,080 | 5,217,129/ 181,705 | 5,175,203 | 5,135,922 | 5,274,417 |
| GC content (%) | 43.5 | 35.65 | 35.64/31.62 | 35.60 | 35.60 | 35.70 |
| Contig | 1 | 1 | 2 | 1 | 1 | 1 |
| Genes (total) | 4,536 | 5,191 | 5,270 | 5,019 | 4,951 | 5,109 |
| CDSs (total) | 4,448 | 5,072 | 5,150 | 4,900 | 4,831 | 4,990 |
| Genes (coding) | 4,237 | 4,874 | 5,020 | 4,796 | 4,759 | 4,912 |
| CDSs (with protein) | 4,237 | 4,874 | 5,020 | 4,796 | 4,759 | 4,912 |
| Genes (RNA) | 211 | 119 | 120 | 119 | 120 | 119 |
| rRNAs (5S, 16S, 23S) | 10, 10, 10 | 11, 11, 11 | 11, 11, 11 | 11, 11, 11 | 11, 11, 11 | 11, 11, 11 |
| tRNAs | 86 | 82 | 83 | 82 | 83 | 82 |
| ncRNAs | 5 | 4 | 4 | 4 | 4 | 4 |
| Pseudo Genes (total) | 182 | 198 | 130 | 104 | 72 | 78 |

*^*^*Genome of *N. circulans* GN03 contains one chromosome (5,217,129 bp) and a plasmid (181,705 bp).

**Supplementary Table S2. Specific primers for measuring of H3 and RD1 population in rhizosphere soil.**

| **Name** | **Primer sequence (5' -> 3')** | **Primer length (bp)** | **Product size (bp)** |
| --- | --- | --- | --- |
| H3_*rpoD*_F | CGC GAA GGC GAA ATC GAA AT | 20 | 484 |
| H3_ *rpoD*_R | CAC GCT CTA CCA GTA CCT CG | 20 |  |
| RD1_ *rpoD*_F | GCA AAG CGT ATA TTG CAG GGT | 21 | 470 |
| RD1_ *rpoD*_R | TCG CCT AAA TGA GAA TCA TCT TCT T | 25 |  |

**Supplementary Table S3. Different factors used to test for stimulation of beneficiary RD1 strain.**

| **Media** | **Substrate** | **RD1 growth stimulation** |
| --- | --- | --- |
| King’s B |  | None |
| R2A |  | None |
| M9 |  | None |
| TSA |  | None |
| CPG |  | None |
| LB |  | None |
| NA |  | None |
| R2A | 1% monosaccharides (glucose, fructose, galactose, mannose) | None |
|  | 1% disaccharides (sucrose, lactose, maltose, trehalose) | None |
|  | 1 mM vitamin B (riboflavin, nicotinamide, cyanocobalamin) | None |
|  | 1 % propionate | None |
|  | Catalase from bovine liver (500, 50, 5 U/ml) | None |
|  | High agar concentration (2.5%) | None |
|  | Glass beads | None |
|  | Antibiotics (AMP, KM, GM, CP, TC, STR, ZEO) (5 mg/mL) | None |
|  | NaCl (1%) | None |
|  | Tryptone (1%) | None |
|  | Yeast Extract (0.5%) | None |
|  | Potassium chloride (KCl, 1%) | None |
|  | Media detoxification by H3 | None |
|  | H3 on cellulose membrane filter (0.45 μm, 0.2 μm) | None |
|  | Heat killed H3 or H3+RD1 | None |
| None | Tryptone (1%) + Yeast extract (0.5%) | None |
|  | Soil agar media (100% soil with 1% agar) | None |
| TSA | Skim milk (1%) | None |
|  | Calcium phosphate (0.5%) | None |
|  | Soluble starch (0.2%) | None |
| R2A and R2B | ATP (100 mM, 1 mM, 10 mM) | None |

*AMP, ampicillin; KM, kanamycin; GM, gentamycin; CP, ciprofloxacin; TC, tetracycline; STR, streptomycin; ZEO, zeocin; NaCl, Sodium chloride; KCl, Potassium chloride; ATP, Adenosine triphosphate.

**Supplementary Table S4.** List of 11 ORFs from *P. putida* H3 strain mutant-808, gene function, and homologous genes in Fig. 2c. The homologous sequences were retrieved from NCBI genebank database (https://www.ncbi.nlm.nih.gov/).

| No. ORF | Description | Identity (%) | Organism |
| --- | --- | --- | --- |
| 1 | ABC transporter substrate-binding protein | 97 | *Pseudomonas* sp. 10-1B |
| 2 | TauD/TfdA family dioxygenase | 98 | *Pseudomonas* sp. B14 |
| 3 | LysR family transcriptional regulator | 96 | *Pseudomonas* sp. P21 |
| 4 | TonB-dependent receptor | 98 | *Pseudomonas* sp. Leaf58 |
| 5 | Aryl-sulfate sulfotransferase | 99 | *Pseudomonas* sp. Leaf58 |
| 6 | NADP-dependent succinate-semialdehyde dehydrogenase | 100 | *Pseudomonas* sp. Leaf58 |
| 7 | 4-aminobutyrate--2-oxoglutarate transaminase | 99 | *Pseudomonas* sp. P21 |
| 8 | Response regulator | 94 | *Pseudomonas monteilii* |
| 9 | MULTISPECIES: sensor domain-containing diguanylate cyclase | 95 | *Pseudomonas* |
| 10 | Fatty acid desaturase | 99 | *Pseudomonas* sp. Leaf58 |
| 11 | EAL domoin-containing protein | 96 | Pseudomonas sp. P21 |

**Supplementary Table S5. The List of the top 10 OTUs selected in the random forest model.**

| **#OTUs** | **Taxonomy** |
| --- | --- |
| OTU047 | p__Firmicutes; c__Bacilli; o__Bacillales; f__Bacillaceae; g__Terribacillus; s__Terribacillus_goriensis |
| OTU091 | p__Proteobacteria; c__Gammaproteobacteria; o__Burkholderiales; f__Oxalobacteraceae |
| OTU099 | p__Actinobacteriota; c__Actinobacteria; o__Micrococcales; f__Microbacteriaceae; g__Microbacterium |
| OTU118 | p__Firmicutes; c__Bacilli; o__Bacillales; f__Bacillaceae; g__Bacillus |
| OTU055 | p__Proteobacteria; c__Alphaproteobacteria; o__Rhizobiales; f__Rhizobiaceae; g__Allorhizobium-Neorhizobium-Pararhizobium-Rhizobium |
| OTU039 | p__Actinobacteriota; c__Actinobacteria; o__Streptomycetales; f__Streptomycetaceae; g__Streptomyces |
| OTU098 | p__Firmicutes; c__Bacilli; o__Brevibacillales; f__Brevibacillaceae; g__Brevibacillus |
| OTU113 | p__Bacteroidota; c__Bacteroidia; o__Sphingobacteriales; f__Sphingobacteriaceae; g__Pedobacter; s__Pedobacter_ginsengisoli |
| OTU030 | p__Firmicutes; c__Bacilli; o__Bacillales; f__Bacillaceae; g__Bacillus; s__Bacillus_asahii |
| OTU035 | p__Proteobacteria; c__Alphaproteobacteria; o__Rhizobiales; f__Rhizobiaceae |
